# LAIR1 drives glioma progression by nuclear focal adhesion kinase dependent expressions of cyclin D1 and immunosuppressive chemokines/cytokines

**DOI:** 10.1038/s41419-023-06199-9

**Published:** 2023-10-16

**Authors:** Xiaoqian Wei, Shushan Pan, Zirui Wang, Jieru Chen, Li Lu, Qizhi Cao, Shuling Song, Huachang Zhang, Xiaohui Liu, Xianjun Qu, Xiukun Lin, Huanli Xu

**Affiliations:** 1 Department of Pharmacology, School of Basic Medical Sciences, Capital Medical University, Beijing, P.R. China; 2 Department of Immunology, School of Basic Medical Sciences, Binzhou Medical University, Yantai, Shandong 264003 P.R. China; 3 School of Gerontology, Binzhou Medical University, Yantai, 264003 Shandong P.R. China; 4 College of Marine Sciences, Beibu Gulf University, Qinzhou, 535011 Guangxi P.R. China

**Keywords:** CNS cancer, Translational research

## Abstract

Leukocyte-associated immunoglobulin-like receptor-1 (LAIR1), an immune receptor containing immunoreceptor tyrosine-based inhibiory motifs (ITIMs), has emerged as an attractive target for cancer therapy. However, the intrinsic function of LAIR1 in gliomas remains unclear. In this study, the poor prognosis of glioma patients and the malignant proliferation of glioma cells in vitro and in vivo were found to be closely correlated with LAIR1. LAIR1 facilitates focal adhesion kinase (FAK) nuclear localization, resulting in increased transcription of cyclin D1 and chemokines/cytokines (CCL5, TGFβ2, and IL33). LAIR1 specifically supports in the immunosuppressive glioma microenvironment via CCL5-mediated microglia/macrophage polarization. SHP2^Q510E^ (PTP domain mutant) or FAK^NLM^ (non-nuclear localizing mutant) significantly reversed the LAIR1-induced growth enhancement in glioma cells. In addition, LAIR1^Y251/281F^ (ITIMs mutant) and SHP2^Q510E^ mutants significantly reduced FAK nuclear localization, as well as CCL5 and cyclin D1 expression. Further experiments revealed that the ITIMs of LAIR1 recruited SH2-containing phosphatase 2 (SHP2), which then interacted with FAK and induced FAK nuclear localization. This study uncovered a critical role for intrinsic LAIR1 in facilitating glioma malignant progression and demonstrated a requirement for LAIR1 and SHP2 to enhance FAK nuclear localization.

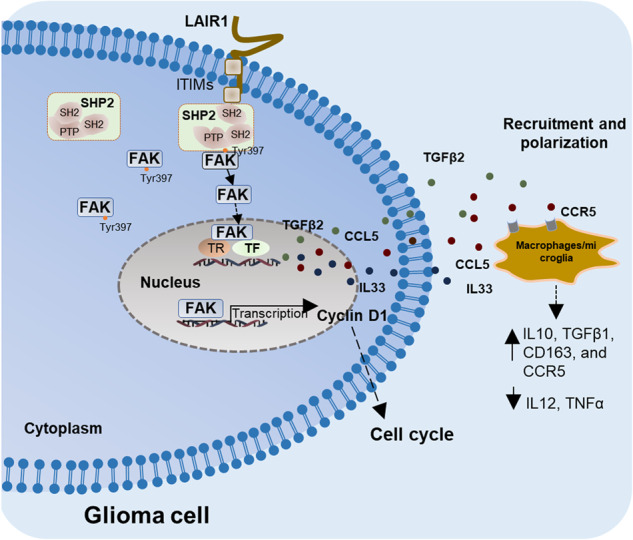

## Introduction

Malignant gliomas are the most common type of primary brain tumor with a high mortality rate. Despite breakthroughs in current conventional therapies for gliomas, the establishment of chemoresistance or relapse frequently leads to treatment failure in most glioma patients [1]. In recent years, immune checkpoint inhibitors, such as programmed cell death protein 1 (PD-1) [2], cytotoxic T-lymphocyte-associated antigen 4 (CTLA-4) [3], and indoleamine 2,3-dioxygenase (IDO) [4, 5], have been successfully used in cancer therapy, thus attracting increased interest in the development of immune checkpoint inhibitors for glioma. However, due to the specific anatomical and physiological characteristics of the brain, current immune checkpoint therapy for gliomas is particularly challenging [6, 7].

Immunoreceptor tyrosine-based inhibitory motifs (ITIMs) are consensus sequences located in the intracellular domains of certain transmembrane receptors that play critical roles in the regulation of immune system [8, 9]. In some tumors, strategies targeting ITIM receptors such as PD-1, CTLA-4, and signal regulatory protein α (SIRPα) have yielded curative results [10, 11]. Nevertheless, low response rates and off-target side effects have hindered their clinical application [4, 5]. In addition, CTLA-4 and PD-1 expression levels were low in brain regulatory T cells (Tregs) of glioma bearing mice [12], indicating that alternative (perhaps glioma-specific) immune checkpoints may play an essential role in immunosuppression of the glioma microenvironment.

Leukocyte-associated immunoglobulin-like receptor 1 (LAIR1, CD305), an immunological checkpoint with a C2-type Ig-like domain and two ITIMs, is widely expressed in immune cells [13, 14]. The two ITIMs of LAIR1 are thought to be the basis of its inhibitory activity [15, 16]. Binding of LAIR1 with its high-affinity ligand collagen can induce autophosphorylation of ITIMs, which then recruit phosphatases such as Src homology phosphotyrosine phosphatase-1 (SHP1) and SHP2 to regulate negative signals [17, 18]. Given the high abundance of collagen in the tumor microenvironment, collagen/LAIR1 signaling in tumor cells plays an important role in downregulating antitumor responses [14]. Inhibition of LAIR1 signaling has been shown to render resistant cancer cells more susceptible to anti-PD-1/PD-L1 therapy [19, 20], indicating that LAIR1 may serve as a potential biomarker for cancer immunotherapy.

In recent years, abnormal LAIR1 expression and various prognostic values have been documented in some cancer types, including leukemia, ovarian cancer, renal carcinoma, oral squamous cell carcinoma, and hepatocarcinoma [14, 21, 22]. However, the expression and function of LAIR1 in glioma have received little attention. Therefore, this study examined the intrinsic oncogenic effect of LAIR1 in glioma.

## Results

### LAIR1 expression in glioma patients was closely correlated with poor prognosis

**Fig. 1:**
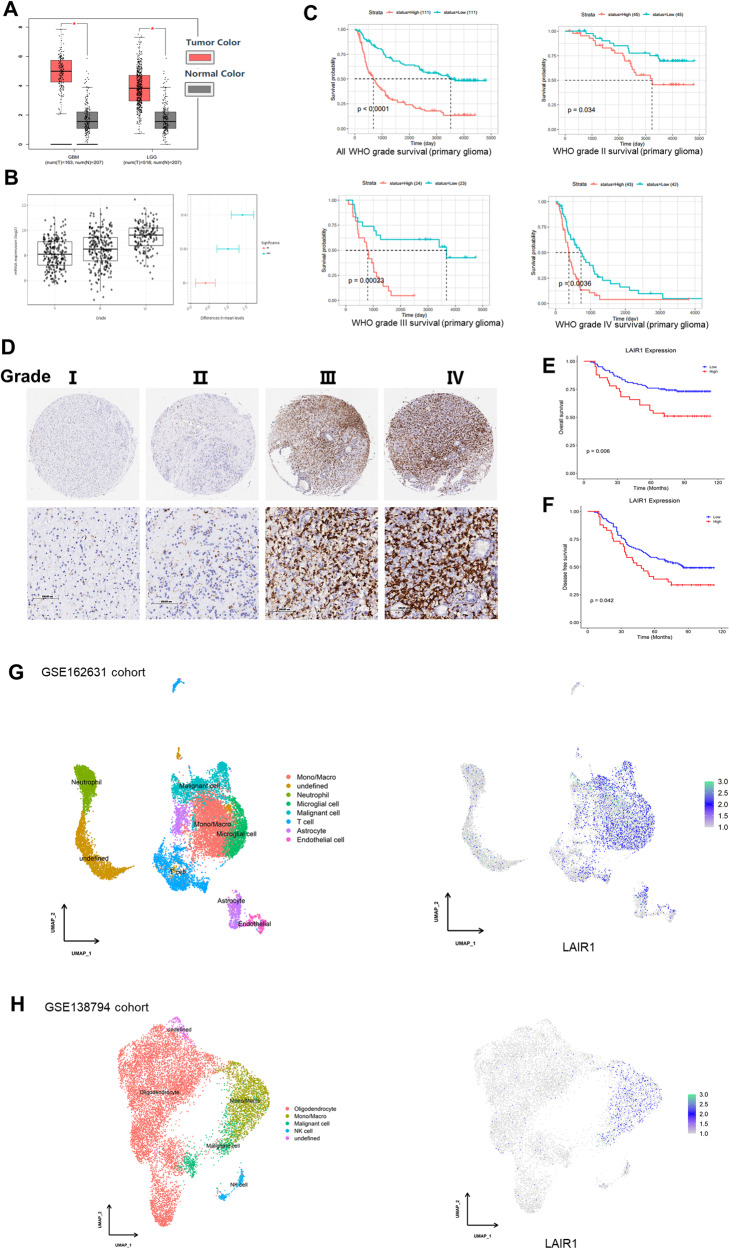
LAIR1 was closely correlated with poor prognosis of glioma patients and promoted the growth of glioma cells. **A** Expression of LAIR1 in GBM and LGG tissues in GEPIA (https://portal.gdc.cancer.gov/). **B** mRNA expression levels of LAIR1 in glioma patients of different WHO grades (From GlioVis: http://gliovis.bioinfo.cnio.es/); **C** The relationship between LAIR1 expression and survival of patients with different WHO glioma grades (Chinese Glioma Genome Atlas database, http://cgga.org.cn/). **D** Representative immunohistochemical staining images for LAIR1 expression in human glioma tissue microarray encompassing 162 glioma patient specimens (HBraG180Su01). **E** The relationship between LAIR1 expression and overall survival of the glioma patients; **F** The relationship between LAIR1 expression and disease-free survival rates of the glioma patients; **G** Single-cell RNA sequencing analysis of LAIR1 expression in GSE162631 cohort. **H** Single-cell RNA sequencing analysis of LAIR1mexpression in GSE138794 cohort.

Bioinformatics analysis revealed that many different types of tumor tissues expressed LAIR1 at higher levels than those in their corresponding normal tissues, particularly in acute myeloid leukemia, esophageal cancer, brain tumor, renal clear cell carcinoma and thymoma (Fig. S1A). Compared to matching normal tissues, the expression of LAIR1 in low-grade glioma (LGG) and glioblastoma (GBM) tissues was notably higher (http://ualcan.path.uab.edu/, Fig. 1A). Additionally, there was a positive correlation between LAIR1 mRNA expression and the WHO glioma grade (Fig. 1B). The expression of LAIR1 was found to be inversely correlated with the overall survival probability of patients with all WHO glioma grades (*P* < 0.0001), WHO grade II glioma (*P* = 0.034), WHO grade III glioma (*P* = 0.00023), and WHO grade IV glioma (*P* = 0.0036) (Fig. 1C).

LAIR1 expression was detected by immunohistochemistry in a human glioma tissue microarray encompassing 162 glioblastoma patient specimens (HBraG180Su01). LAIR1 expression was significantly higher in glioma tissues than in the adjacent normal tissues (Figs. S1B and 1D). Furthermore, LAIR1 expression correlated with WHO grade (*P* = 0.001) and patient age (*P* = 0.009) (Fig. S1C, D, Table S1). Patients with low LAIR1 expression had significantly longer overall and disease-free survival rates than those with high LAIR1 expression (Fig. 1E, F, Table 1).

**Table 1:** Univariate and multivariate analyses of the factors correlated with Overall survival of glioma carcinoma patients.

variables	Univariate analysis				Multivariate analysis			
	*P* value	HR	95% CI		*P* value	HR	95% CI	
			Lower	Upper			Lower	Upper
expression	0.007	2.147	1.227	3.756	0.827	1.066	0.6	1.895
Gender	0.144	1.551	0.861	2.796				
Age	<0.0001	3.31	1.766	6.204	<0.0001	3.377	1.787	6.38
Grade stage	<0.0001	12.501	6.773	23.075	<0.0001	12.675	6.743	23.827

*HR* hazard rate; *CI* confidence interval.

To evaluate whether LAIR1 is expressed in glioma cells, expression of LAIR1 in human brain cancer tissues was analyzed using single-cell RNA sequencing data (GSE162631 and GSE138794). The result showed that LAIR1 was more highly expressed in malignant cells and macrophages/monocytes than in other cell types, including neutrophils, T cells, astrocytes, and endothelial cells (Fig. 1G, H).

### LAIR1 promoted the growth of glioma cells in vitro and in vivo

**Fig. 2:**
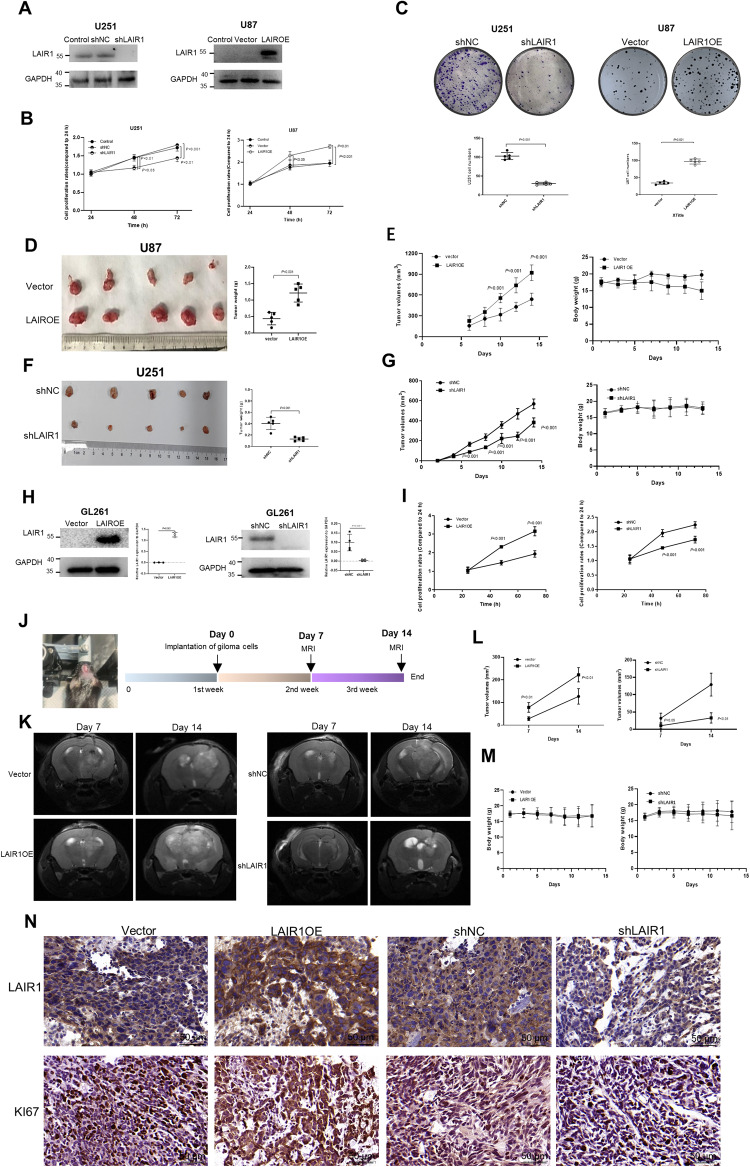
LAIR1 promoted the growth of glioma cells in vivo. **A** LAIR1 expression in LAIR1OE or shLAIR1 glioma cells by Western blot and quantification of protein bands (*n* = 4). **B** The proliferation curves of LAIR1OE or shLAIR1 glioma cells by MTS assay (*n* = 5). **C** The colony formation abilities of LAIR1OE or shLAIR1 glioma cells by colony formation assay and quantification (*n* = 5). Nude mice were subcutaneously injected with LAIR1OE U87 cells or shLAIR1 U251 cells and their corresponding control cells (*n* = 6). **D**, **F** The images and weights of excised tumors 14 days after subcutaneous implantation of LAIR1OE U87 cells and shLAIR1 U251 cells in nude mice (*n* = 5). **E**, **G** The tumor volumes and body weights of the mice after implantation of LAIR1OE U87 cells and shLAIR1 U251 cells (*n* = 5). **H** Expression of LAIR1 in LAIR1OE (*n* = 3) or shLAIR1 GL261 (*n* = 4) cells detected by Western blot and quantification of protein bands. **I** The proliferation curves of LAIR1OE or shLAIR1 GL261 cells by MTS assay (*n* = 5). **J** Schematic of the formation of the orthotopic transplantation model in mice. **K** MRI images of the tumors in the brains of the mice on days 7th and 14th following implantation (*n* = 6). **L** Tumor volumes in the brains of the mice bearing LAIR1OE or shLAIR1 GL261 cells (*n* = 6). **M** Body weights of the mice bearing LAIR1OE or shLAIR1 GL261 cells (*n* = 6). **N** Immunohistochemical staining for LAIR1 and KI67 in the glioma tissues of the mice bearing LAIR1OE or shLAIR1 GL261 cells (*n* = 6). The data were expressed as average ±SD. *P* values were labeled in each figure.

RT-PCR and Western blot were used to detect LAIR1 expression in six glioma cell lines (human U87, U251, T98G, and U138 cells, mouse GL261 cells, and rat C6 cells). LAIR1 was found to be significantly expressed in U251 and T98G cells, but not in U87 or C6 cells, as shown in Fig. S2A, B. Thus, LAIR1 overexpression cell lines (LAIR1OE) were established using U87 and C6 cells, whereas knockdown experiments were conducted using U251 and T98G cells (shLAIR1). The effectiveness of LAIR1 overexpression or knockdown in these cells was confirmed by Western blot (Figs. 2A and S2C). Overexpression of LAIR1 considerably increased the proliferation and colony formation abilities of U87 and C6 cells, whereas knockdown of LAIR1 significantly decreased the proliferation and colony formation abilities of T98G and U251 cells (Figs. 2B, C, S2D, E).

The in vivo growth of glioma cells with different LAIR1 expression was studied in nude mice using a subcutaneously transplanted tumor model. In vivo growth of shLAIR1 U251 cells was significantly reduced, while that of LAIR1OE U87 cells was significantly increased compared to their corresponding control cells, as shown in Fig. 2D–G. There were no significant differences in body weights between the groups (Fig. 2E, G).

GL261 glioma cells stably overexpressing (LAIR1OE) or knocking down (shLAIR1) LAIR1 were successfully constructed to establish an orthotopic glioma model (Fig. 2H). MTS assay showed that the expression of LAIR1 significantly promoted the proliferation of GL261 cells in vitro (Fig. 2I). Fig. 2J shows a schematic of the establishment of an orthotopic mouse glioma model. Magnetic resonance imaging was used to examine the proliferation of GL261 cells in the brains of mice on days 7th and 14th following implantation. The results showed that the tumor volumes of mice bearing LAIR1OE cells were significantly larger than those of mice bearing vector cells (Fig. 2K, L), whereas those of mice bearing shLAIR1 cells were significantly smaller than those of mice bearing control cells (Fig. 2K, L). In addition, no significant differences in body weight were observed between the groups (Fig. 2M). Immunohistochemical staining of LAIR1 and KI67 in LAIR1OE tissues was evidently stronger than that of neighboring normal tissues and vector tissues, whereas that of shLAIR1 tissues was noticeably weaker than in adjacent normal tissues and control tissues (Fig. 2N). These results suggest that LAIR1 plays an important role in promoting glioma cell growth in vivo.

### LAIR1 promoted the cell cycle progression of glioma cells

**Fig. 3:**
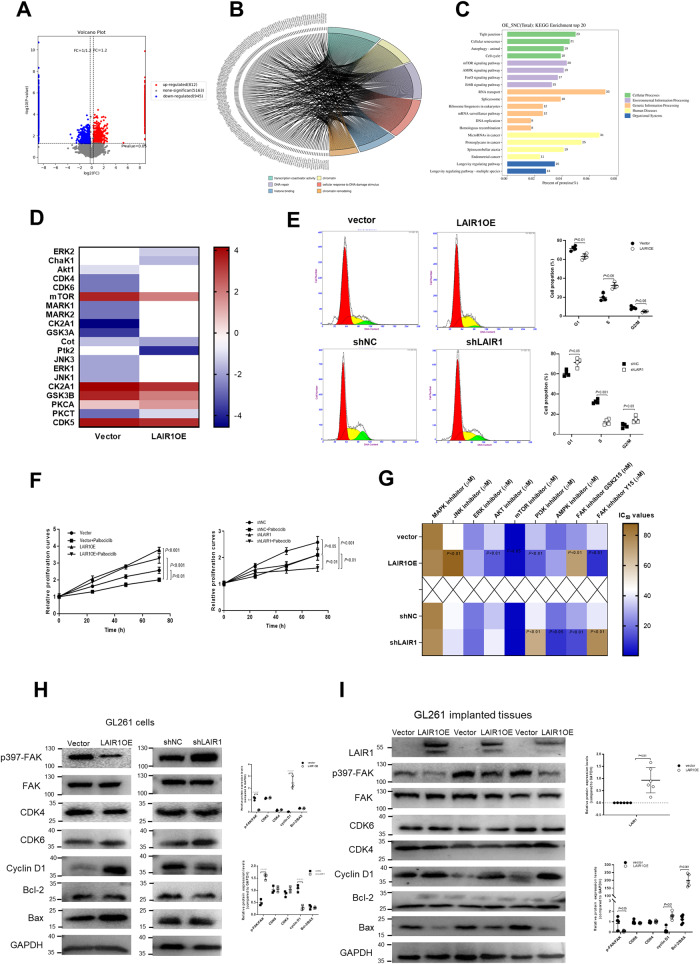
LAIR1 promoted the cell cycle progression. **A** The volcano plot of differentially phosphorylated proteins (criteria: fold change ≥1.2, *P* < 0.05) (*n* = 3). **B** Chord diagram showing the relationship between the selected GO keywords and the differential sites of corresponding proteins (*n* = 3). **C** KEGG enrichment analysis bubble map of the first 20 differentially expressed pathway (*n* = 3). **D** The most significantly changed upstream kinases by upstream kinase prediction analysis (*n* = 3). **E** Cell cycle analysis of LAIR1OE or shLAIR1 GL261 cells by PI staining and statistical analysis (*n* = 4). **F** Relative growth rates of LAIR1OE and shLAIR1 GL261 cells in the presence of 0.5 μM Palbociclib for 24 h, 48 h, and 72 h (*n* = 3). **G** Heatmap showing the IC_50_ values of different inhibitors in LAIR1OE and shLAIR1 GL261 cells MTS assay (*n* = 3). SB203580: MAPK inhibitor; SP600125: JNK inhibitor; PD98059: ERK inhibitor; MK2206: AKT inhibitor; INK-128: mTOR inhibitor; HY-101115: PI3K inhibitor; BML275: AMPK inhibitor; Y15: FAK inhibitor; GSK215: FAK inhibitor. **H** Protein expressions in LAIR1OE and shLAIR1 GL261 cells by Western blot and quantification of protein bands (*n* = 3). **I** Protein expressions in LAIR1OE GL261 cells implanted tissues by Western blot and quantification of protein bands (*n* = 6). The data were expressed as average ±SD. *P* values were labeled in each figure.

Since LAIR1 can regulate the phosphorylation or dephosphorylation of many downstream molecules [23, 24], phosphoproteomic analysis was performed using LAIR1OE and vector cells. As shown in Fig. S3A, 812 up-regulated sites and 945 down-regulated sites were identified in LAIR1OE cells compared to vector cells. A volcano plot of differentially phosphorylated proteins is shown in Fig. 3A (criteria: fold change ≥1.2, *P* < 0.05). The phosphorylated proteins were classified into three major groups using gene ontology (GO) annotation (Fig. S3B). The chord map showed the relationship between the selected GO keywords and the differential sites of the corresponding proteins (Fig. 3B), implying that LAIR1 overexpression induced significant alterations in nuclear events. According to Kyoto Encyclopedia of Genes and Genomes (KEGG) analysis, the most significantly modified pathways included those associated with the cell cycle, MTOR signaling pathway, and the AMPK signaling pathway (Fig. 3C). Ptk2 (also called focal adhesion kinase, FAK) and CDK4/6 were among the most significantly altered kinases according to upstream kinase prediction (Fig. 3D).

The cell cycle and apoptosis were assessed to study the role of LAIR1 in the growth of glioma cells. PI staining demonstrated that LAIR1 knockdown did cause G1 phase cell cycle arrest (Fig. 3E). Additionally, compared to vector cells, the CDK4/6 inhibitor palbociclib demonstrated lower sensitivity to LAIR1OE cell growth and higher sensitivity to shLAIR1 cell growth (Fig. 3F). These findings revealed that LAIR1 aids glioma growth through cell cycle regulation. Although annexin V/PI staining revealed no influence of LAIR1 on in vitro apoptosis (Fig. S3C), TUNEL labeling revealed obvious in vivo apoptosis in shLAIR1 GL261 glioma tissues (Fig. S3D). Furthermore, the apoptotic inhibitor Z-VAD-FMK had no discernible effect on the proliferation of LAIR1OE, shNC, or shLAIR1 cells in vitro (Fig. S3E).

To evaluate the role of altered kinases in the growth of glioma cells, IC_50_ values of related phosphorylation pathway inhibitors were determined in LAIR1OE, shLAIR1, and their corresponding control cells (Fig. 3G). It is noteworthy that FAK inhibitor (GSK215, but not Y15) showed more obvious growth inhibitory effects on shLAIR1 cells than on control and LAIR1OE cells (Fig. 3G), indicating that FAK may be involved in modulating LAIR1-related signaling pathways. Based on these results, Western blot analysis showed that LAIR1 significantly decreased the expression of p397-FAK/FAK, while increasing the expression of cyclin D1 in LAIR1OE cells (Fig. 3H). An opposite expression pattern was observed in shLAIR1 cells. Significantly decreased p397-FAK/FAK and increased cyclin D1 expression were also found in the LAIR1OE glioma tissues of mice (Fig. 3I). In addition, the increased Bcl-2/Bax ratio was only found in LAIR1OE tissues of mice, but not in LAIR1OE GL261 cells (Fig. 3H, I), further implying that LAIR1 may trigger apoptosis resistance only in vivo.

### LAIR1 regulated the cell cycle via nuclear FAK-dependent cyclin D1 expression

**Fig. 4:**
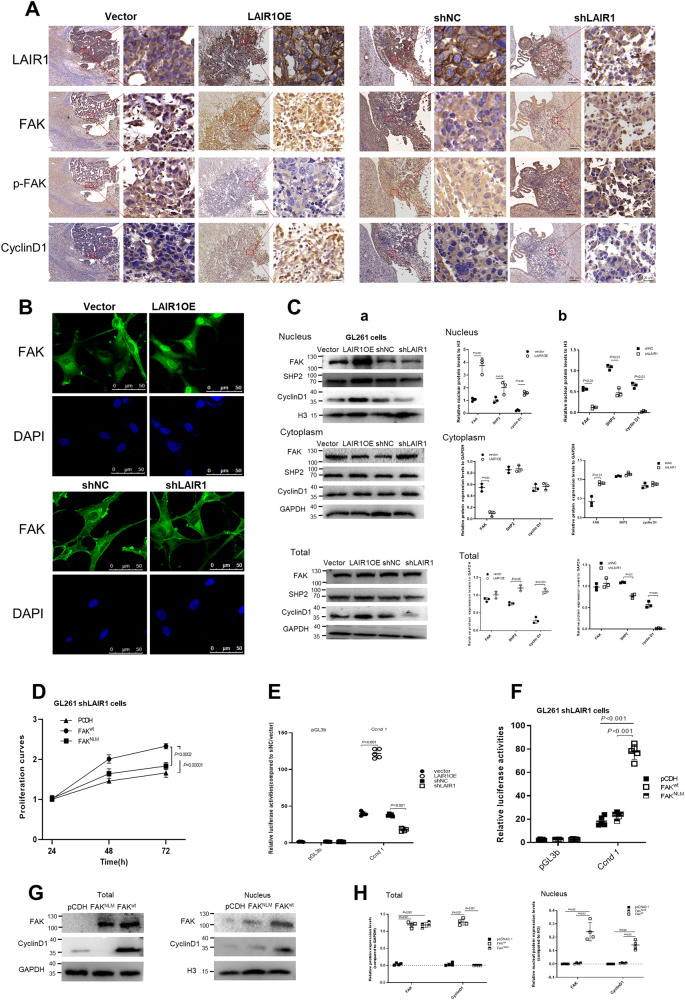
LAIR1 induced nuclear FAK-dependent cyclin D1 expression. **A** Immunohistochemical staining for LAIR1, FAK, p-FAK, and cyclin D1 in serial sections of the glioma tissues of mice bearing LAIR1OE or shLAIR1 GL261 cells (*n* = 6). **B** Immunofluorescence staining for FAK in LAIR1OE and shLAIR1 GL261 cells (*n* = 3). **C** Nuclear, cytoplasmic, and total FAK, SHP2, and cyclin D1 expressions in LAIR1OE and shLAIR1 GL261 cells by Western blot (a) and quantification of protein bands (b) (*n* = 3). **D** Effects of FAK^wt^ and FAK^NLM^ on cell proliferations of shLAIR1 GL261 cells by MTS assay (*n* = 3). **E** The transcriptional activities of the *Ccnd1* promoter in LAIR1OE or shLAIR1 GL261 cells by luciferase reporter assay (*n* = 5). **F** The transcriptional activities of the *Ccnd1* promoter in shLAIR1 GL261 cells co-transfected with FAK^wt^ and FAK^NLM^ (*n* = 5). **G**, **H** FAK and cyclin D1 expressions in the nuclear and total cells of shLAIR1 GL261 cells co-transfected with FAK^wt^ and FAK^NLM^ by Western blot (a) and quantification (b) (*n* = 4). The data were expressed as average ±SD. *P* values were labeled in each figure.

FAK typically functions in a kinase-dependent manner. However, recent studies have indicated that FAK can also translocate to the nucleus and regulate the transcription of several oncogenes [25, 26]. Additionally, reduced p397-FAK expression has been linked to FAK nuclear localization [27]. As p397-FAK was shown to be considerably reduced in LAIROE cells (Fig. 3), nuclear FAK expression was detected. The results demonstrated that FAK had an apparent nuclear localization in LAIR1OE tissues but not in control or shLAIR1 tissues (Fig. 4A). Immunofluorescence staining and Western blot analysis showed that LAIR1 significantly increased the nuclear localization of FAK in LAIR1OE GL261 cells (Fig. 4B, C). Increased nuclear FAK expression was also examined in human glioma cells to confirm that the nuclear localization of FAK is not exclusive to mouse glioma cells. Similar results were found in LAIR1OE U87 cells but not in shLAIR1 U251 cells (Fig. S4A, B).

To further investigate whether nuclear FAK is necessary for glioma cell growth, wild-type FAK (FAK^wt^) and FAK nonnuclear localizing mutant (FAK^NLM^) constructs were created and transfected into shLAIR1 GL261 cells. As shown in Fig. 4D, induction of FAK^wt^ promoted the growth of shLAIR1 GL261 cells, whereas that of FAK^NLM^ showed much weaker effects. FAK promotes cell cycle progression by enhancing transcriptional activation of the cyclin D1 promoter [28]. Because LAIR1 significantly increased the protein levels of cyclin D1 (Figs. 3H, I, 4C, and S4), the transcriptional activity of *Ccnd1* in LAIR1OE and shLAIR1 GL261 cells was determined using a luciferase reporter gene assay. The result showed that overexpression of LAIR1 significantly increased the transcriptional activity of the *Ccnd1* promoter in LAIR1OE cells (Fig. 4E). FAK^wt^, but not FAK^NLM^, significantly increased the transcriptional activity of *Ccnd1* promoter in shLAIR1 GL261 cells (Fig. 4F). Furthermore, Western blot analysis revealed that FAK^wt^, but not FAK^NLM^, significantly increased nuclear and total cyclin D1 protein levels (Fig. 4G, H). These findings revealed that the cell cycle regulatory effects of LAIR1 were mediated by nuclear FAK-dependent cyclin D1 expression.

### LAIR1 induced nuclear FAK-dependent production of C-C chemokine ligand 5 (CCL5), interleukin-33 (IL33), and transforming growth factor beta2 (TGFβ2)

**Fig. 5:**
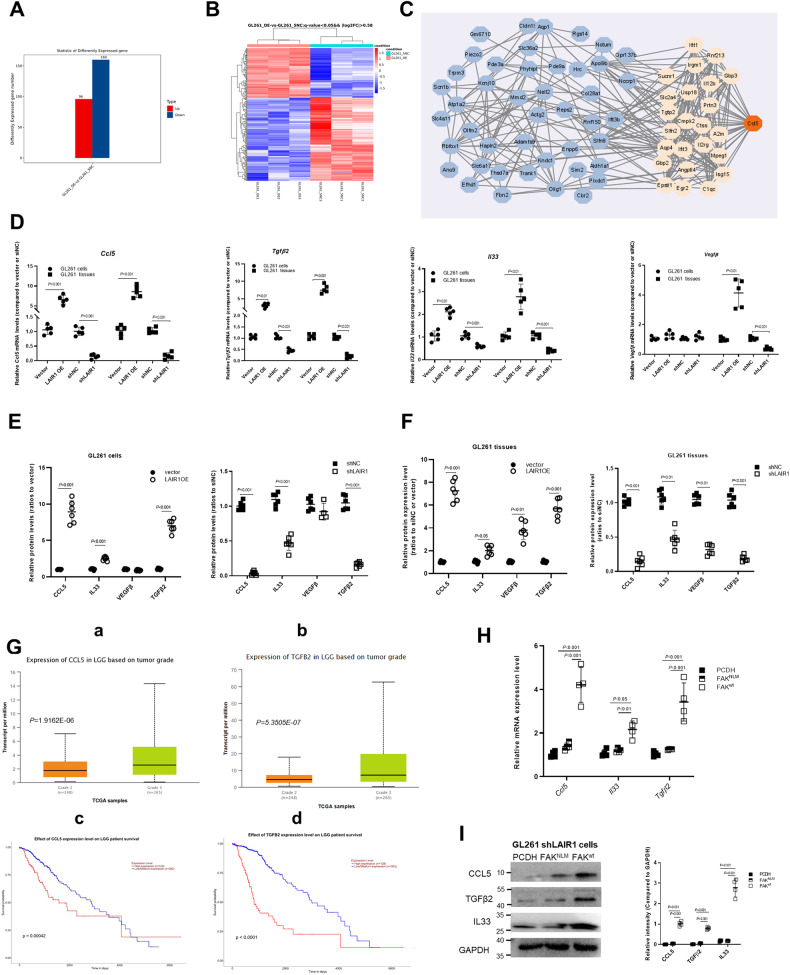
LAIR1 induced FAK-dependent production of CCL5, IL33, and TGFβ2. **A** Statistical histogram of differentially expressed genes of LAIR1OE and shNC GL261 cells by RNA-sequencing analysis. O: LAIR1OE cells, NC: vector cells (*n* = 3). **B** The distribution of up-regulated and down-regulated differential genes by heatmap (criteria:| log2FC | ≥0.58, *P* < 0.05). **C** PPI subnetwork of CCL5. **D** *Ccl5*, *Tgfβ2*, *Il33*, and *Vegfβ* mRNA expressions in LAIR1OE or shLAIR1 GL261 cells and implanted tissues by RT-PCR (*n* = 5). **E** CCL5, TGF-β2, IL33, and VEGFβ expressions in LAIR1OE or shLAIR GL261 cells by ELISA (*n* = 6). **F** CCL5, TGFβ2, IL33, and VEGFβ expressions in LAIR1 OE or shLAIR1 GL261 tissues by ELISA (*n* = 6). **G** CCL5 and TGFβ2 expression levels in LGG patients (a,b) and their relationships with survival (c,d) in TCGA (From http://ualcan.path.uab.edu/). **H** *Ccl5*, *Tgfβ2*, *Il33*, and *Vegfβ* mRNA levels in shLAIR1 GL261 cells co-transfected with FAK^wt^ and FAK^NLM^ by RT-PCR (*n* = 4). **I** CCL5, IL33, and TGFβ2 expressions in shLAIR1 GL261 cells co-transfected with FAK^wt^ and FAK^NLM^ by Western blot and quantification (*n* = 4). The data were expressed as average ±SD. *P* values were labeled in each figure.

RNA sequencing analysis was performed to further explore the mechanism by which LAIR1 promotes the malignant progression of gliomas. Compared with vector cells, 96 genes were significantly up-regulated and 160 genes were significantly down-regulated in LAIR1OE GL261 cells (Fig. 5A). The overall distribution of the differentially expressed genes is shown in Fig. 5B. GO and KEGG analyses of the differentially expressed genes are shown in Fig. S5A, B. Interestingly, CCL5 showed a key regulatory role in the protein–protein interaction (PPI) network constructed using differentially expressed genes (Fig. 5C).

Nuclear FAK has been shown to promote tumor progression by regulating the transcription of cytokines/chemokines, including CCL5, TGFβ2, IL33, and VEGFβ, which in turn drive the establishment of an immunosuppressive microenvironment [29, 30]. CCL5, TGFβ2, IL33, and vascular endothelial growth factor β (VEGFβ) expression in LAIR1OE and shLAIR1 GL261 cells and their corresponding implanted tissues were detected. RT-PCR showed that mRNA expression levels of *Ccl5*, *Il33*, and *Tgfβ2* were significantly upregulated in LAIR1OE cells and their corresponding implanted tissues, whereas those of *Ccl5*, *Il33*, and *Tgfβ2* were significantly down-regulated in shLAIR1 cells and their corresponding implanted tissues (Fig. 5D). ELISA showed that LAIR1 significantly increased CCL5, IL33, and TGFβ2 protein levels in GL261 cells and implanted tissues (Fig. 5E, F). These results showed that LAIR1 enhanced the production of CCL5, TGFβ2, and IL33 both in vitro and in vivo. The changes in *Vegfβ* mRNA and protein levels in implanted tissues were more noticeable than those in GL261 cells (Fig. 5D–F), indicating that VEGFβ may be secreted by surrounding cells but not by glioma cells.

Bioinformatics analysis revealed that the expression of CCL5, TGFβ2, VEGFβ, and IL33 was much higher in both LGG and GBM tissues than in the corresponding normal tissues (http://ualcan.path.uab.edu/, Figs. 5G and S6A–F), and was closely correlated with prognosis. To determine whether the increased production of CCL5, IL33, and TGFβ2 was nuclear FAK-dependent, the mRNA and protein levels of *Ccl5*, *Il33*, and *Tgfβ2* were detected in shLAIR1 GL261 cells transfected with FAK^wt^ and FAK^NLM^ constructs using RT-PCR and Western blot. The results showed that the production of CCL5, IL33, and TGFβ2 was significantly enhanced by FAK^wt^ but not by FAK^NLM^ (Fig. 5H, I). These results suggest that LAIR1 induces the nuclear FAK-dependent production of CCL5, IL33, and TGFβ2.

### LAIR1 modulated microglia/macrophage polarization partially by CCL5

**Fig. 6:**
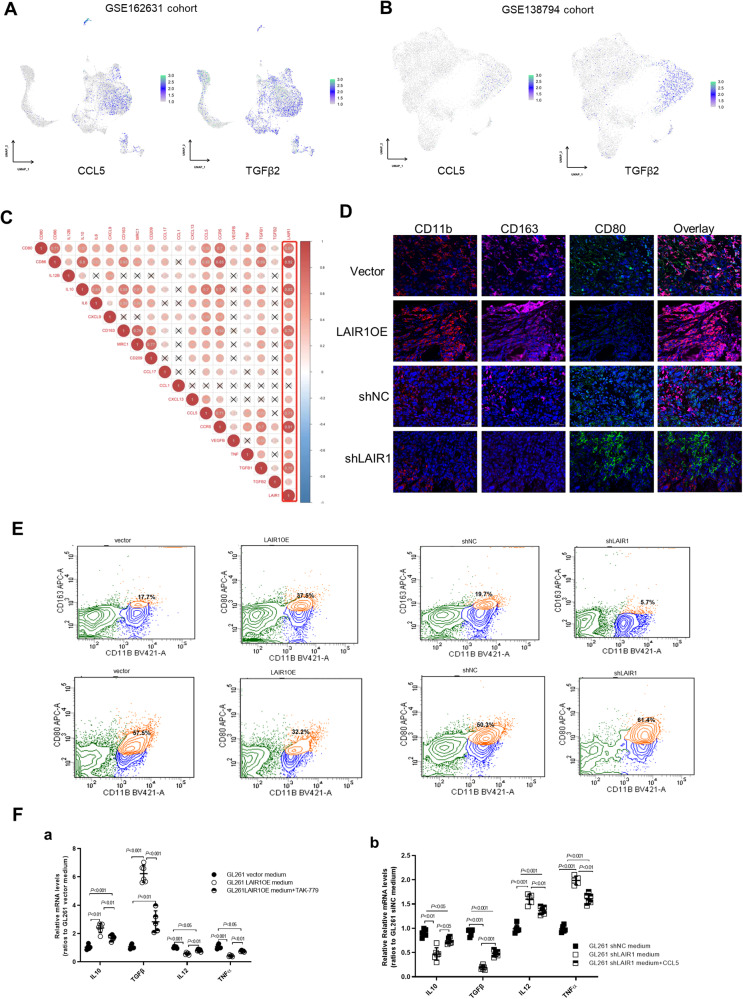
LAIR1 induced CCL5 production and modulated microglia/macrophage polarization. **A** Single-cell RNA sequencing analysis of CCL5 and TGFβ2 expression in GSE162631 cohort. **B** Single-cell RNA sequencing analysis of CCL5 and TGFβ2 expression in GSE138794 cohort. **C** Spearman correlation analysis between LAIR1 and microglia/macrophage makers of glioblastoma samples from the GTEx database (http://xena.ucsc.edu/public/) by R (version 4.1.2). **D** Representative images of LAIR1OE or shLAIR1 implanted tissues stained for binding of CD11b (red), CD163 (pink), and CD80 (green) antibodies. DAPI (blue) was used for nuclear staining. Scale bars: 50 μm (*n* = 3). **E** FACS quantification for percentage of CD163^+^/CD11b^+^ and CD 80^+^/CD11b^+^ in LAIR1OE or shLAIR1 implanted tissues (*n* = 3). **F** Relative mRNA expression levels of *Il10*, *Tgfβ*1, *Il12*, and *Tnfα* in BV2 cells cultured in the medium of LAIR1OE or shLAIR1 GL261 cells in the presence of CCL5 (10 ng/mL) or CCR5 antagonist TAK-779 (1.2 nM) for 48 h by RT-PCR (*n* = 5). The data were expressed as average ±SD. *P* values were labeled in each figure.

Understanding the role of LAIR1 in glioma immunology is crucial because the majority of receptors harboring ITIMs are involved in immune system regulation. Using single-cell RNA sequencing data (GSE162631 and GSE138794), we found that LAIR1, CCL5, and TGFβ2 was more highly expressed in malignant cells and macrophages/monocytes than in other cell types (Figs. 1G, H and 6A, B). Using glioma samples from the GTEx database, Spearman correlation analysis of LAIR1 expression and macrophage markers was performed. As shown in Fig. 6C, LAIR1 expression was positively correlated with many M2 microglia/macrophage markers, especially CD86, IL10, CD163, CD206 (MRC1), CCL5, C-C chemokine receptor type 5 (CCR5), and TGFβ1, in LGG and GBM tissues. Then, multiple staining for CD11b, CD163, and CD80 was performed in LAIR1OE- or shLAIR1-implanted tissues. As shown in Fig. 6D, increased CD163 (pink) and decreased CD80 (green) expression were found in the LAIR1OE-implanted tissues, whereas decreased CD163 (pink) and increased CD80 (green) expression were found in the shLAIR1-implanted tissues, compared with the corresponding control groups. The flow cytometry result also showed that the percentage of CD163^+^/CD11b^+^ was significantly increased in LAIR1OE GL261 tissues compared to the vector tissues, and decreased in the shLAIR1-implanted tissues (Fig. 6E). The percentage of CD80^+^/CD11b^+^ levels was reversed (Fig. 6E). These results demonstrate that LAIR1 controls microglia/macrophage polarization to influence the immune response in gliomas.

To further investigate the role of LAIR1 in microglia/macrophage polarization, BV2 cells were cultured in the medium of LAIR1OE or shLAIR1 GL261 cells. The RT-PCR result showed that the M2 microglia/macrophage markers *Il10* and *Tgfβ1* were significantly increased in BV2 cells cultured in the medium of LAIR1OE cells and decreased in BV2 cells cultured in the medium of shLAIR1 cells, whereas the M1 microglia/macrophage markers *Il12* and *Tnfα* were reversed (Fig. 6F). Western blot analysis showed that the expression levels of M2 microglia/macrophage markers CD163 and CCR5 were significantly increased in BV2 cells cultured in LAIR1OE medium and decreased in BV2 cells cultured in shLAIR1 medium (Fig. S7A, B). The CCL5/CCR5 axis plays an important role in establishing an immunosuppressive tumor microenvironment [31, 32]. These microglia/macrophage makers were further detected in BV2 cells cultured in conditioned media in the presence of CCL5 or the CCR5 antagonist, TAK-779. TAK-779 reduced the elevated expression of *Il10*, *Tgfβ1*, CD163, and CCR5 in BV2 cells cultured in LAIR1OE medium, whereas CCL5 rescued the expression of *Il12* and *Tnfα* in BV2 cells cultured in LAIR1OE medium (Figs. 6F and S7). The opposite effects were observed in BV2 cells cultured in shLAIR1 medium (Figs. 6F and S7). These results suggest that CCL5 plays an important role in LAIR1-induced microglia/macrophage polarization.

### ITIMs/LAIR1 and PTP/SHP2 were required for the nuclear localization of FAK

**Fig. 7:**
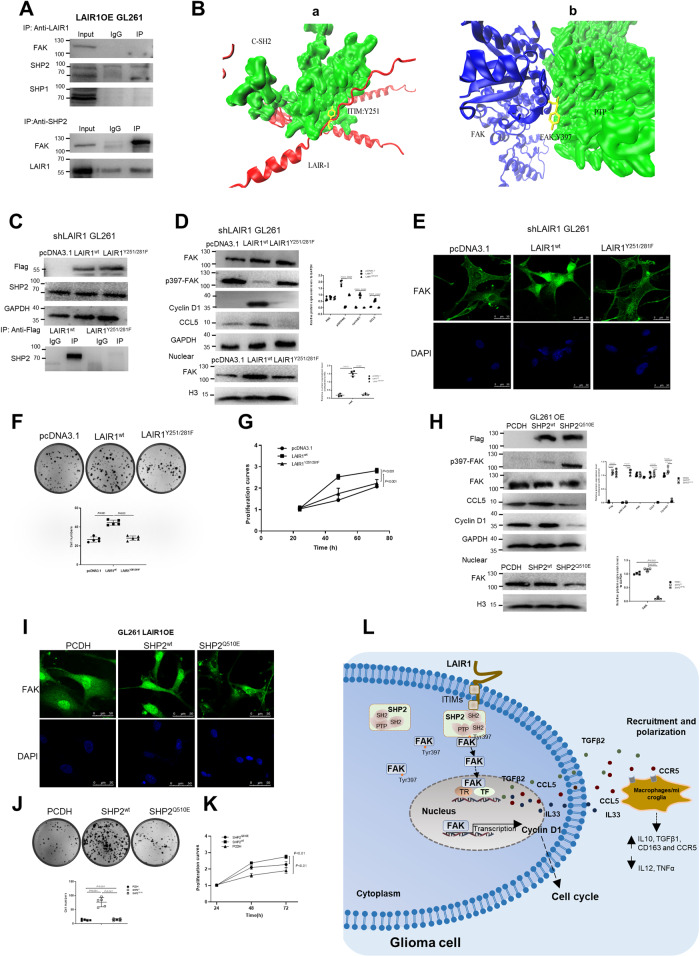
ITIMs/LAIR1 and PTP/SHP2 were required for the nuclear localization of FAK. **A** LAIR1–SHP2 and SHP2–FAK interactions in LAIR1OE GL261 cells by co-immunoprecipitation. Normal rabbit IgG antibody was used as control. *n* = 3. **B** LAIR1–SHP2 (a) and SHP2–FAK (b) docking result. LAIR1 was displayed in red, SHP2 in green, and FAK in blue. **C** Effects of LAIR1^wt^ and LAIR1^Y251/281F^ mutant on LAIR1–SHP2 interaction by co-immunoprecipitation (*n* = 3). **D** FAK, p-FAK, cyclin D1, and CCL5 expressions in shLAIR1 GL261 cells expressing LAIR1^wt^ or LAIR1^Y251/281F^ constructs by Western blot and quantification (*n* = 4). **E** Immunofluorescence staining for FAK in shLAIR1 GL261 cells expressing LAIR1^wt^ or LAIR1^Y251/281F^ constructs (*n* = 3). **F** Colony formation abilities of shLAIR1 GL261 cells expressing LAIR1^wt^ or LAIR1^Y251/281F^ constructs by colony formation assay (*n* = 5). **G** Proliferation of shLAIR1 GL261 cells expressing LAIR1^wt^ or LAIR1^Y251/281F^ constructs by MTS (*n* = 5). **H** FAK, p-FAK, cyclin D1, and CCL5 expression in GL261 OE cells expressing SHP2^wt^ or SHP2^Q510E^ constructs by Western blot and quantification (*n* = 4). **I** Immunofluorescence staining for FAK in LAIR1OE GL261 cells expressing SHP2^wt^ or SHP2^Q510E^ constructs (*n* = 3). **J** Colony formation abilities of GL261 LAIR1OE cells expressing SHP2^wt^ or SHP2^Q510E^ constructs by colony formation assay (*n* = 5). **K** Proliferation of LAIR1OE GL261 cells expressing SHP2^wt^ or SHP2^Q510E^ constructs by MTS (*n* = 5). **L** Schematic of how LAIR1 drives glioma growth and macrophage immunosuppression. The data were expressed as average ±SD. *P* values were labeled in each figure.

The ITIMs of LAIR1 can recruit phosphatases such as SHP1 and SHP2 [33]. The direct interaction of LAIR1–SHP2, but not LAIR1–SHP1, was verified by a co-immunoprecipitation assay (Fig. 7A). Furthermore, SHP2 clearly interacted with FAK (Fig. 7A). We speculated that LAIR1 might induce the nuclear localization of FAK by interacting with SHP1 or SHP2, which then recruits FAK. Molecular docking results showed that SHP2 bound to the ITIMs of LAIR1 (red) via its C-SH2 domain (green), exposing the PTP domain that could interact with the Y397-containing FAK interface (blue), leading to FERM-mediated autoinhibition of FAK (Fig. 7B).

To confirm whether the ITIMs of LAIR1 are essential for its interaction with SHP2, LAIR1^wt^ and LAIR1^Y251/281F^ mutant (Y251/281F point mutation in the ITIMs) constructs were transfected into shLAIR1 GL261 cells. The co-immunoprecipitation assay showed that the LAIR1–SHP2 interaction was identified in LAIR1^wt^/shLAIR1 GL261 cells, but was almost completely absent in LAIR1^Y251/281F^/shLAIR1 GL261 cells (Fig. 7C), suggesting that the ITIMs of LAIR1 are essential for the LAIR1/SHP2 complex. Furthermore, Western blot and immunofluorescence staining results showed that the LAIR1^wt^ construct significantly increased nuclear FAK levels in shLAIR1 GL261 cells, whereas the LAIR1^Y251/281F^ mutation did not significantly affect nuclear FAK levels (Fig. 7D, E). Additionally, cyclin D1 and CCL5 expression, cell viability, and the clonogenic formation ability of shLAIR1 GL261 cells were significantly increased by LAIR1^wt^ but not by LAIR1^Y251/281F^ (Fig. 7D–G). In addition, decreased p397-FAK expression was found in LAIR1^wt^/shLAIR1 GL261 cells, but not in LAIR1^Y251/281F^/shLAIR1 GL261 cells (Fig. 7D).

To evaluate whether the nuclear localization of FAK was dependent on SHP2, LAIR1OE GL261 cells stably expressing wild type SHP2 (SHP2^wt^) and a loss-of-function mutant (SHP2^Q510E^) were established. The findings demonstrated that in LAIR1OE GL261 cells, SHP2^Q510E^ significantly increased p397-FAK levels and reduced FAK nuclear localization (Fig. 7H, I). The expression of cyclin D1 and CCL5, as well as the viability and capacity for clone formation of LAIR1OE GL261 cells, were significantly lowered by the SHP2^Q510E^ mutant (Fig. 7H, J, K). These results showed that the nuclear localization of FAK was dependent on the ITIMs of LAIR1 and the PTP of SHP2.

## Methods

### Materials and reagents

The chemicals, reagents, and antibodies used in this study are shown in Supplemental Table S2.

### Bioinformatic analysis

Pan cancer view of LAIR1 expression was obtained from the Cancer Genome Atlas (TCGA) using the http://ualcan.pathuab.edu web portal. Based upon data generated by TCGA, China brain Glioma Genome Atlas (CGGA) (http://cgga.org.cn/) and GTEX (https://gtexportal.org), further processing was performed using GlioVis (http://gliovis.bioinfo.cnio.es/) and GEPIA (https://portal.gdc.cancer.gov/) to conduct bioinformatics analysis. The relationships between LAIR1 and co-expressed genes were analyzed using the Cox regression model in the R package. The results were presented in heatmap using “reshape2” and “R Color Brewer” of R package.

Single-cell RNA sequencing analysis was performed to explore the abundance of LAIR1 in different immune cell types in the glioma tissues. The GSE138794 and GSE162631 cohorts were downloaded from the Gene Expression Omnibus (GEO) repository and single-cell RNA sequencing analysis was performed using the Seurat package of R software. Genes expressed in less than three cells or with less than 200 unique gene counts were excluded. Then, “vst” of Seurat was used to pick the top 2000 highly variable genes. The UMAP algorithm is used to reduce the dimensionality of the data. Cell clusters were annotated using the Cell Maker datasets (http://xteam.xbio.top/CellMarker/).

### Immunohistochemical staining of glioma tissue chip

A human glioma tissue chip (HBraG180Su01) containing 162 glioblastoma specimens was purchased from Shanghai Outdo Biotech Co., Ltd., China. After routine dewaxing, hydration, and heat-induced antigen retrieval, endogenous enzymes in the specimens were inactivated with 3% H_2_O_2_ in methanol for 20 min. The sections were blocked with 5% bovine serum albumin (BSA) for 1 h and then incubated with LAIR1 antibody (1:500) at 4 °C overnight. After washing with PBS, sections were incubated with HRP-conjugated goat anti-rabbit IgG for 45 min at room temperature. LAIR1 expression was visualized by DAB staining. The chip was scanned using a pannoramic scanner (3DHISTECH Ltd. Hungary).

### Construction of LAIR1 overexpressing or knocking-down glioma cell lines

Human glioma cell lines (U251, T98G, and U87), a rat glioma cell line (C6), and a mouse glioma cell line (GL261) were obtained from the Cell Resource Center of Peking Union Medical College, Beijing, China. All the cell lines were tested for mycoplasma contamination and authenticated using short tandem repeat profiling. Lentiviral vector LV5 was used to construct LAIR1 overexpressing recombinant lentivirus (LAIR1OE, GenePharma, Suzhou, China). LAIR1OE and vector constructs were transfected into U87, C6, and GL261 cells according to the manufacturer’s instructions. Twenty-four hours after transfection, stable cell clones were screened using complete DMEM containing 4 μg/mL puromycin. pFU-GW-016-shRNA-LAIR1 lentivirus (sequences shown in supplementary Table S3, Genechem, Shanghai, China) was used to construct stable LAIR1 knocking-down U251, T98g, and GL261 cells. Twenty-four hours after transfection, cells were cultured with 4 μg/mL puromycin for screening.

### Cell proliferation assay and colony formation assay

For the cell proliferation assay, cells were seeded in 96-well plates and cell viability was detected by MTS assay using the CellTiter 96® AQueous One Solution Cell Proliferation Assay kit (Promega, Madison, WI, USA) according to the manufacturer’s instructions. For the colony formation assay, cells were seeded in 35 mm culture dishes (200 cells/dish) and cultured under normal conditions to allow colony formation (approximately 2 weeks). The cell colonies were stained with 0.1% crystal violet and counted under an inverted microscope.

### Cell cycle assay

After overnight serum starvation, LAIR1OE, shLAIR1, and their corresponding control cells were collected and fixed in 70% ethanol at 4 °C for 24 h. After washing with PBS for 2 times, the cells were stained with PI/RNase solution (50 μg/mL PI and 200 μg/mL RNase) for 30 min. The cell cycle stage was detected by flow cytometry and analyzed using FlowJo software.

### In vivo glioma growth experiment

BALB/c-nu/nu nude mice (4–6 weeks, 18–22 g) were purchased from Beijing Weitonglihua Experimental Animal Technology Co., Ltd., Beijing, China. The mice were randomly divided into four groups (*n* = 6). LAIR1 overexpressing or knocking-down cells were collected and subcutaneously inoculated as previously described [33]. The tumor volumes and body weights of the mice were measured every alternate day. Two weeks later, all mice were sacrificed and the tumors were dissected and weighed. The investigator was blinded to the groups during the evaluation. and the mouse with no obvious tumor growth was excluded.

The orthotopic transplantation model of GL261 cells was established using C57BL/6 mice (male, 4–6 weeks, 18–22 g), as previously described [33]. The mice were randomly divided into four groups (*n* = 8). After rapid anesthesia with isoflurane, the head of the mouse was fixed on a stereotactic instrument. LAIR1OE or shLAIR1 GL261 cells were injected using a microsyringe. Seven and 14 days after implantation, intracranial tumors were detected using a Bruker 7.0 T Micro-MRI system (Bruker BioSpec 70/20 USR, German). The mouse with a maximum tumor diameter of >2.0 ± 0.5 mm was considered successful. At the end of the experiment, all mice were sacrificed and their brain tissues were excised. The investigator was blinded to the groups during the evaluation. Brain tissues were further used for Western blot, ELISA, hematoxylin-eosin (HE) staining and immunohistochemical staining.

### Imunohistochemical staining

Brain tissues were fixed in 4% formalin, embedded in paraffin, and cut into 4–6 μm thick sections. The sections were subjected to antigen retrieval and endogenous enzyme inactivation after deparaffinization and rehydration. The sections were then blocked with 5% BSA and incubated with primary antibodies. After washing, the sections were incubated with HRP-conjugated anti-rabbit IgG and visualized by DAB staining. The sections were reviewed and scored by two pathologists. All stained images were recorded using a high-capacity digital slide scanner system (3DHISTECH Ltd., Budapest, Hungary).

An Opal 4-Color Manual IHC Detection Kit (PerkinElmer, NEL810001KT) was used for multiplex staining. After deparaffinization, hydration, antigen unmasking, and quenching, slides were incubated with primary antibodies (Anti-CD11b: 1:200, Anti-CD163: 1:200, Anti-CD80: 1:150, Anti-CCR5: 1:200), secondary polymer antibodies, and fluorophore-conjugated TSA. Subsequently, staining and detection steps were performed with a different tyramide-fluorophore conjugate. Nuclei were counterstained with DAPI. Slides were scanned and quantified using a Zeiss Axio Scan.Z1 and Zen Blue software (Carl Zeiss Microscopy GmbH, Göttingen, Germany).

### RT-PCR

Total RNA was extracted using TRIzol reagent and reverse-transcribed to cDNA using the Revert Aid First Strand cDNA Synthesis Kit (Thermo Fisher Scientific Inc., MA, USA). RT-PCR was performed using a DyNAmo ColorFlash SYBR Green qPCR kit (Thermo Fisher Scientific Inc., Waltham, MA, USA). The primers used are listed in Supplementary Table S3. GAPDH was used as an internal control. The relative fold-changes in the target genes were calculated using the comparative Ct method.

### Total and nuclear protein extraction

For total protein extraction, cells were collected and resuspended in RIPA buffer containing a protease and phosphatase inhibitor cocktail (Sigma Aldrich, MO, USA) for 30 min on ice. Supernatants were obtained by centrifugation at 12000 rpm for 15 min. Nuclear proteins were extracted using a Nuclear Protein Extraction Kit (Solarbio Science & Technology Co., Ltd., Beijing, China). Approximately 3 × 10^7^ cells were collected and resuspended in Buffer A 10 min to destroy the cell membrane. Nuclear membranes were digested with Buffer B for 60 min on ice. After centrifugation, the supernatant was transferred to a dialysis bag and incubated in overnight Buffer C at 4 °C. The final supernatant was collected by centrifugation at 12,000 rpm and 4 °C for 30 min. Protein concentrations were determined using the BCA Protein Assay Kit (Sangon Biotech Co., Ltd., Shanghai, China).

### Western blot

Total and nuclear protein extracts were separated by sodium dodecyl sulfate polyacrylamide gel electrophoresis (SDS-PAGE) and transferred to polyvinylidene fluoride (PVDF) membranes. After blocking with 5% bovine serum albumin (BSA) at room temperature for 2 h, the membranes were probed with primary antibodies at 4 °C overnight. After washing, the membranes were incubated with the corresponding secondary antibodies for 1 h. Finally, the membranes were visualized using an ECL Chemiluminescent Substrate Reagent Kit (Merck Millipore Ltd., USA) according to the manufacturer’s instruction.

### Label-free quantitative phosphoproteomics

LAIR1OE GL261 cells and vector cells (passage 3) were harvested and label-free quantitative phosphoproteomics was performed by Shanghai Luming Biotechnology Co., Ltd. (Shanghai, China). Briefly, total protein was extracted and protein concentration was determined. The peptides were then subjected to trypsin treatment and peptide desalination. After the enrichment and purification of the phosphorylation-modified peptides, the samples were analyzed using an Orbitrap Fusion mass spectrometer equipped with a Nanospray Flex source (Thermo Fisher Scientific Inc., USA). The parameters were as follows: C18 column (25 cm × 75 µm); flow rate: 300 nL/min; mobile phase A: 0.1% FA in water, B: 80% ACN/0.1% FA in water; linear gradient: 0 ~ 66 min, 3%–27% B; 66 ~ 73 min, 27%–46% B; 73 ~ 84 min, 46%–100% B; 84 ~ 90 min, 100% B; electrospray voltage: 1.5 KV; mass range: 100–1700 m/z; mass resolution: 60,000; AGC target: 4e5. Dynamic exclusion: 40.0 s. Raw data were analyzed using the MaxQuant software (njs-0.7.6 version, Thermo Fisher Scientific Inc. USA). The database search was performed with the following parameters: Instrument type: Bruker TIMS; Fixed modifications: Carbamidomethyl(C); Variable modification: Oxidation (M), Acetyl (Protein N-term), phosphor (STY); First search peptide tolerance: 20 ppm; Main search peptide tolerance: 10 ppm; MS/MS match tolerance: 0.5 Da; Max.Missed: 2. The peptide spectrum matches were filtered using Percolator (PD 2.1) to adjust the 1% FDR. ptmRS33 (PD 2.1) was used to obtain localization probability score (PC) for each putatively modified site. Phosphopeptides with PC ≥ 25 were used for the analysis.

### RNA sequencing analysis

LAIR1OE GL261 cells and vector cells (passage 3) were harvested and total RNA was extracted using the TRIzol reagent. RNA purity, integrity, and concentration were evaluated, and qualified RNA samples were used to construct RNA sequencing libraries. Libraries were sequenced using an Illumina HiSeq X-ten sequencing System. Clean reads were obtained by removing low-quality reads using the Trimmomatic software. The clean reads were mapped to GRCH38 (https://asia.ensembl.org/info/data/ftp/index.html) using the HISAT2. The read counts of each gene were obtained using the HTSeq count. Differentially expressed genes were analyzed using the DESeq R package. The significantly differentially expressed genes were selected using *P* value < 0.05, and fold change > 2 or fold change < 0.5. The differentially expressed genes in LAIR1OE and vector cells were defined using hierarchical cluster analysis. GO enrichment analysis was performed using the DAVID suite (http://david.abcc.ncifcrf.gov/tools.jsp). KEGG pathway mapping was performed using R based on hypergeometric distribution. PPI were analyzed using Cytoscape 3.9.1 software.

### Generation of targeting constructs

Wild type LAIR1 (LAIR1^wt^) and the Y251/281 F mutant in the ITIMs of LAIR1 (LAIR1^Y251/281F^) were constructed by GenePharma (Shanghai, China). The DNA sequences encoding LAIR1^wt^ and LAIR1^Y251/281F^ were confirmed by automated DNA sequencing and cloned into the pcDNA3.1. Transfection efficiency of LAIR1 in shLAIR1 GL261 cells was confirmed by western blot. FAK^NLM^ (R177A/R178A) was constructed using the Agilent QuikChange^TM^ Site-Directed Mutagenesis Kit as described previously [34]. The DNA sequences encoding FAK^wt^ or FAK^NLM^ were amplified by PCR and ligated into the pCDH-CMV-MCS-EF1-Neo vector using DpnI restriction enzyme. Primer sequences are listed in Supplementary Table S3. Lentivirus constructs were transfected into shLAIR1 GL261 cells using Lipofectamine 3000. Twenty-four hours after transfection, stable cell clones were screened in complete DMEM containing 4 μg/mL puromycin and 4 μg/mL G418. The DNA encoding residues Met1-Leu525 of mouse SHP2 were inserted into a pCDH-CMV-EF1-Neo vector. The SHP2^Q510E^ mutant was constructed by site-directed mutagenesis [35]. After DNA sequencing and comparison with the NCBI database (NM_011202, Swiss-Prot: P35235.2), constructs were inserted into the pCDH-CMV-EF1-Neo vector. Primer sequences are listed in Supplementary Table S3. The lentivirus constructs were transfected into GL261 LAIR1OE cells. Twenty-four hours after transfection, stable cell clones were screened in complete DMEM containing 4 μg/mL puromycin and 4 μg/mL G418.

### Luciferase reporter gene assay

The mCCND1 promoter segment (5’sequence) was amplified by PCR and inserted into the pGL3-basic vector to generate the luciferase construct as described previously [29]. The construct was further identified using double enzyme digestion and DNA sequencing. The luciferase reporter plasmid was transfected into LAIR1OE, and shLAIR1 GL261 cells or co-transfected into GL261 shLAIR1 cells along with pCDH vectors encoding FAK^wt^ and FAK^NLM^ mutants. Two days after transfection, luciferase activity was determined using a dual-luciferase Reporter Gene Assay system (Promega, USA). All assays were performed in triplicate.

### Co-immunoprecipitation assay

A Co-immunoprecipitation assay was performed using the BeaverBeads™ Protein A/G Immunoprecipitation Kit according to the manufacturer’s protocol. Briefly, the primary antibody was incubated with the magnetic beads at room temperature with gentle rotation for 20 min. After washing with a magnetic rack, cell lysates were added and incubated with gentle rotation at 4 °C overnight. After washing, the immunoprecipitated complex was collected, resuspended in 1 × loading buffer, and boiled at 100 °C for 5 min. The samples were then analyzed by western blot.

### ELISA

Cells and glioma tissues were collected, lysed, and centrifuged at 12000 rpm for 20 min at 4 °C. The supernatants were then collected and the corresponding protein levels were detected using a mouse CCL5 ELISA Kit (SEKM-0043), mouse TGFβ2 ELISA kit (SEKM-0036), mouse IL33 ELISA kit (SEKM-0028), and mouse VEGFβ ELISA kit (SEKM-0039) according to the manufacturer’s instructions. The absorbance measurements were detected using a microplate reader (GloMax® Discover Microplate Reader, Promega, WI, USA).

### Molecular docking study

The LAIR1/SHP2/FAK interaction was studied using molecular docking [36]. LAIR1 (UniPort ID: Q6GTX8) and SHP2 (UniPort ID: Q06124) were used as receptor and ligand molecules, respectively, to obtain a complex by GRAMM (global ridge molecular matching). The complex was used as the receptor molecule and FAK (UniPort ID: Q00944) as a ligand molecule to study their interactions. The LAIR1/SHP2/FAK complex was then analyzed using PDBePISA (https://www.ebi.ac.uk/msd-srv/prot_int/pistart.html) and rendered usingy VMD (version 1.9.3).

### Flow cytometry

Mouse glioma tissues were harvested, mechanically dissociated, and digested in DMEM containing collagenase/dispase (Roche) at 37 °C for 30 min. Tumor cells were resuspended in FACS buffer and then incubated with FcR at 4 °C for 20 min. Cells were then incubated with the indicated fluorescent antibodies listed in the Table S2. Data were collected by flow cytometry (FACSCalibur, BD, USA).

### Statistical analysis

All data are expressed as the mean ± S.D. of at least three independent experiments. *n* values ae shown in the corresponding figure legends. Statistical significance was analyzed using two-tailed Student’s *t* test or one-way analysis of variance. All statistical tests were performed using the GraphPad Prism software (version 8.0; GraphPad Software, San Diego, CA, USA).

## Discussion

Different LAIR1 expressions and predictive values have been found in several cancer types in recent years [21, 37, 38]. However, the precise function of LAIR1 in gliomas remains unknown. In the current investigation, we discovered that LAIR1 was significantly correlated with the malignant proliferation of glioma cells both in vitro and in vivo, as well as with the poor overall survival of glioma patients. Interestingly, we found that ITIMs of LAIR1 could recruit SHP2 and induce nuclear localization of FAK, resulting in increased expression of cyclin D1, CCL5, IL33, and TGFβ2. Specifically, LAIR1 partially contributes to the immunosuppressive glioma microenvironment by CCL5-mediated microglia/macrophage polarization. These results shed light on the possible role of the LAIR1/SHP2/FAK axis as a promising target for glioma treatment (Fig. 7L).

FAK is a nonreceptor protein tyrosine kinase that promotes the development and progression of cancer [39, 40]. FAK typically functions as a cytoplasmic kinase and can be phosphorylated at Tyr397 in response to integrin or growth factor receptor signaling [41]. FAK also penetrates the nucleus and regulates several transcriptional networks, affecting cell cycle progression, senescence, and immune evasion. Although the function of nuclear FAK remains poorly characterized, its effects on cyclin D1 transcription and cell cycle regulation have been extensively investigated [31, 42, 43]. Our study also showed that LAIR1 promotes cell cycle progression and increases the transcription and expression of cyclin D1, which is nuclear FAK-dependent (Figs. 3 and 4). FAK dephosphorylation may increase its nuclear translocation according to recent research [44]. In this study, reduced p397-FAK expression and enhanced nuclear translocation of FAK were observed in LAIR1OE cells (Figs. 3, 4, and S4). FAK overexpression has been documented in various tumor types, and FAK inhibitors that reduce FAK expression or activity are currently being investigated for cancer treatment [45]. Our results imply that, at least in glioma cells, LAIR1-induced FAK nuclear translocation has a greater effect on malignant glioma growth than FAK catalytic activity inhibition, emphasizing the importance of assessing LAIR1 expression levels when utilizing FAK inhibitors for cancer treatment.

Increasing evidence has emerged in recent years, showing that nuclear FAK is crucial for creating an immunosuppressive tumor microenvironment [30, 46]. Serrels et al. showed that nuclear FAK increased the expression of many chemokines and cytokines including CCL1, CCL5, CCL7, CXCL10, and TGFβ2, which recruit Tregs and promote CD8^+^ T cell-mediated antitumor response [32]. Nuclear FAK also enhances the gene expression of IL33 and interacted with IL33 to promote transcription of the ST2 receptor, which inhibits the activation of CD8^+^ T cells [30]. In this study, we also showed that LAIR1 remarkably promoted the production of CCL5, TGFβ2, and IL33 in glioma cells, in a nuclear FAK-dependent manner. These findings suggest that LAIR1 is another intrinsic molecule that induces FAK nuclear localization, offering a novel mechanism for its involvement in the immunosuppressive tumor microenvironment.

Macrophages/microglia constitute the dominant tumor-infiltrating immune cells in the immunosuppressive environment of gliomas [47, 48]. Glioma cells can recruit macrophages/microglia and induce their polarization to tumor-supportive M2-macrophages by secreting cytokines such as TGF-β and VEGF, which then facilitate immune escape, invasion, and proliferation of glioma cells. Although the effects of LAIR1 signaling on T cell exhaustion and suppression have been well explored, our findings suggest that LAIR1 is more closely associated with the immunosuppressive polarization of macrophages/microglia in gliomas (Fig. 6C–F). Numerous studies have demonstrated that the CCL5/CCR5 axis is important for cancer growth, migration, and establishment of an immunosuppressive tumor microenvironment [49, 50]. Our study showed that CCL5 induced by intrinsic LAIR1 expression in glioma cells plays an important role in microglia/macrophage polarization, although more research is needed to determine the relationship in other cell types.

ITIMs of LAIR1 can interact with SH2 domain-containing phosphatases such as SHP1 and SHP2, resulting in LAIR1-mediated inhibitory function [26]. However, the SH2 domain-containing phosphatases that can interact with the ITIMs of LAIR1 are tissue-specific [51]. Our results showed that LAIR1 directly interacted with SHP2, but not SHP1, in GL261 cells. SHP2 is a nonreceptor protein oncogenic tyrosine phosphatase that contains one PTP catalytic domain, two tandem SH2 domains, and a C-terminal tail [52, 53]. Typically, the SH2 domain binds to the PTP domain to block phosphatase activity. Upon stimulation, the active region of PTP is exposed after dissociation from SH2, resulting in SHP2 activation [35]. Herein, decreased p397-FAK expression and direct interaction of SHP2 with FAK were found in LAIR1OE cells, showing that the exposed PTP domain of active SHP2 interacted with the FAK interface harboring Y397, resulting in FERM-mediated autoinhibition and nuclear localization of FAK. Additionally, the well-established mutant SHP2^Q510E^ at position 510 in the PTP domain inhibited FAK nuclear localization, demonstrating that the PTP domain of the SHP2 domain is necessary for FAK nuclear localization in gliomas. Recently, there have also been reports of nuclear SHP2 expression, which was most likely caused by the cell density sensing Hippo signal [51]. The reason may be that the Hippo signaling pathway is activated by the increased cell density of LAIR1OE GL261 cells. Future research should concentrate on how the SHP2–FAK interactome may alter its subcellular position, as well as the potential biological and clinical implications of this alteration.

Our study revealed the critical role of intrinsic LAIR1 in promoting the malignant progression of gliomas and established the need for ITIMs/LAIR1 and PTP/SHP2 to promote the nuclear localization of FAK. These results shed light on the potential of the LAIR1/SHP2/FAK axis as an effective therapeutic target for gliomas. The expression and function of LAIR1 in various glioma cell types, and how these cells communicate to control the immunosuppressive microenvironment, should be further investigated.

### Supplementary information

Supplemental data

Original WB

aj-checklis

## Data Availability

All source data for all relevant figures and supplementary figures are available upon reasonable request from the corresponding author. Full and uncropped western blots are uploaded as supplemental material. The raw data of RNA sequencing has been deposited in the National Center for Biotechnology Information (NCBI) (SRA, SUB12982566), and that of quantitative phosphoproteomics has been deposited to the ProteomeXchange Consortium (Identifier: PXD042932) via the iProX partnerrepository.
