# Supplementary material for: LAIR1 drives glioma progression by nuclear focal adhesion kinase dependent expressions of cyclin D1 and immunosuppressive chemokines/cytokines
Source: Cell Death Dis. 2023 Oct 16;14(10):684. doi: 10.1038/s41419-023-06199-9 (PMC10579300; doi:10.1038/s41419-023-06199-9)
Supplement: Supplementary file 1 — Supplemental data [file 41419_2023_6199_MOESM1_ESM.docx]

**Supplemental Figures**


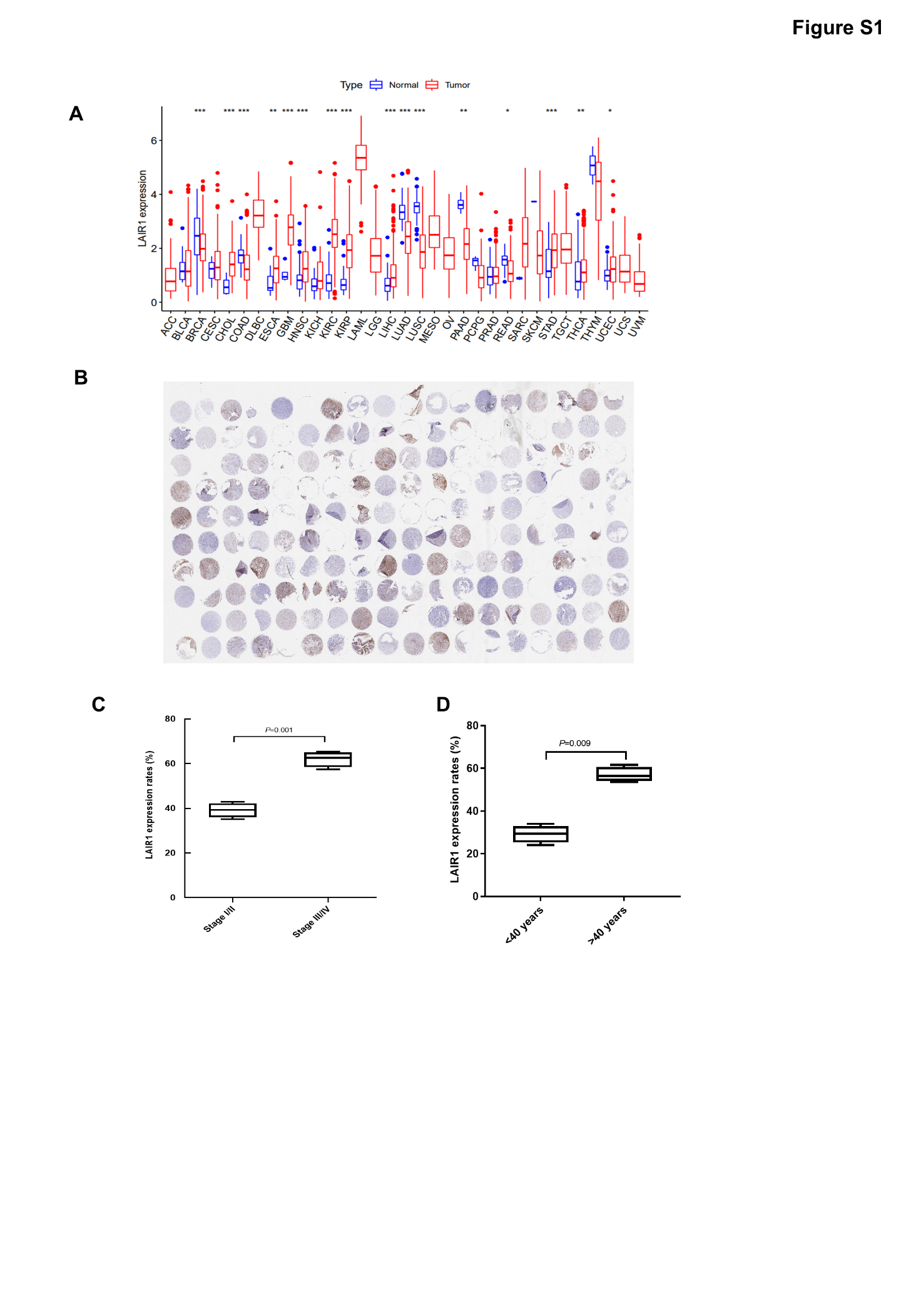


**Fig. S1:** (A) The expression profile of LAIR1 in different types of tumor tissues in TCGA database (From http://ualcan.path.uab.edu/). (B) Immunohistochemical staining results for LAIR1 expression in human glioma tissue microarray encompassing 162 glioma patient specimens (HBraG180Su01). (C) The relationship between LAIR1 expression and WHO grades of the glioma patients; (D) The relationship between LAIR1 expression and ages of the glioma patients.


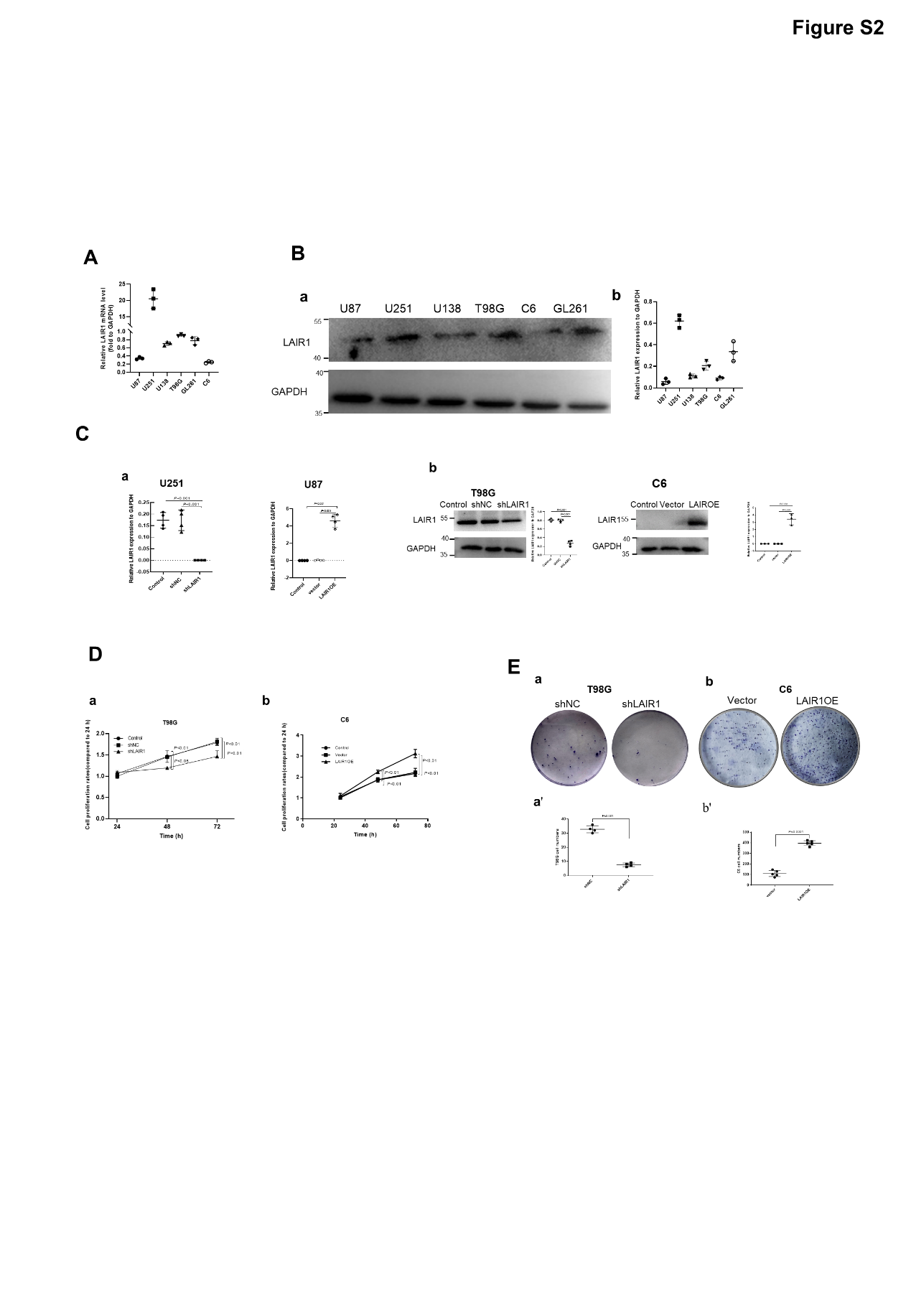


**Fig. S2**: (A) Relative mRNA levels of LAIR1 in different glioma cell lines by qRT-PCR (*n*=3). (B) The expression profile of LAIR1 in different glioma cell lines by Western blot (a) and quantification of protein bands (b) (*n*=3). (C) LAIR1 expression in LAIR1OE (*n*=4) or shLAIR1 (*n*=3) glioma cells by Western Blot (a,b) and quantification of protein bands (a',b'). (D) The proliferation curves of LAIR1OE or shLAIR1 glioma cells by MTS assay (*n*=4). (E) The colony formation abilities of LAIR1OE or shLAIR1 glioma cells by colony formation assay (a,b) and quantification (a',b') (*n*=4).


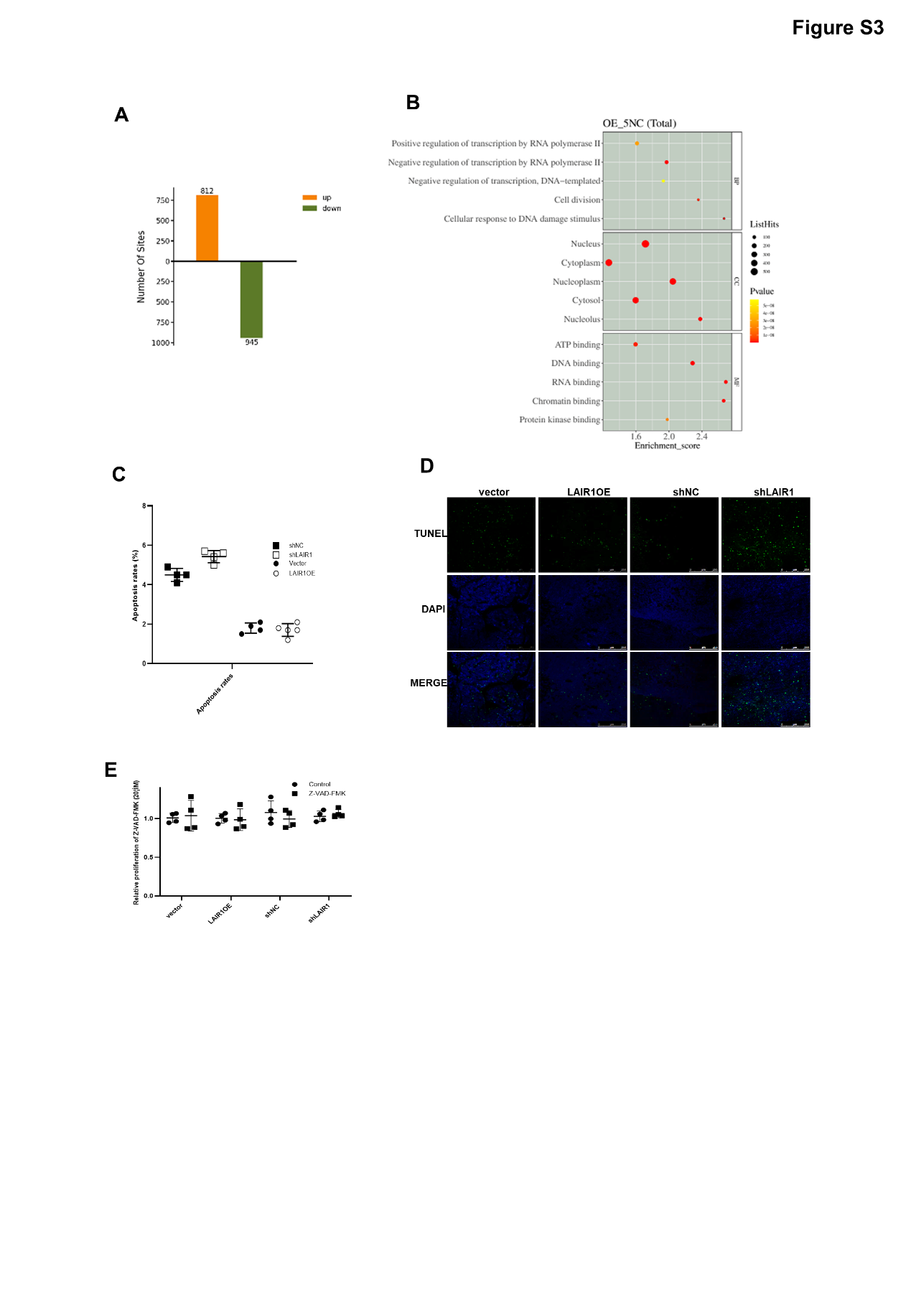


**Fig. S3:** (A) Differentially phosphorylated protein sites in LAIR1OE GL261 cells compared with control cells (*n*=3). (B) GO enrichment classification of differentially expressed genes (*n*=3). (C) Apoptosis rates of LAIR1OE or shLAIR1 glioma cells by Annex V/PI staining (*n*=5). (D) TUNEL staining of the LAIR1OE or shLAIR1 glioma tissues (*n*=6). (E) Relative growth rates of LAIR1OE and shLAIR1 GL261 cells in the presence of 20 μM Z-VAD-FMK (*n*=4). The data were expressed as average ±SD.


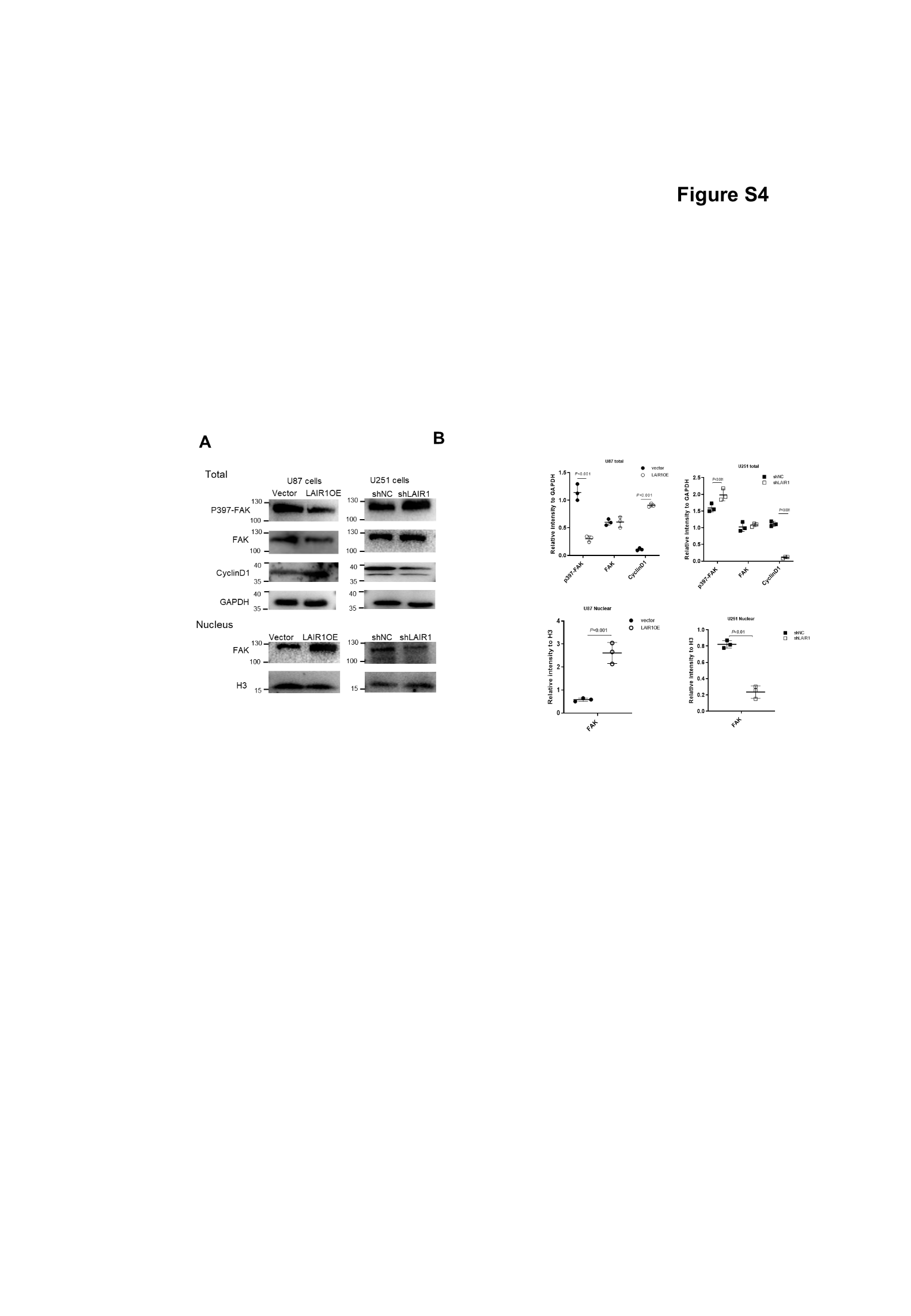


**Fig. S4:** FAK, p-FAK, SHP2, and cyclin D1 expressions in LAIR1OE U87 cells and shLAIR1 U251 cells by Western blot (A) and quantification of protein bands (B) (*n*=3).


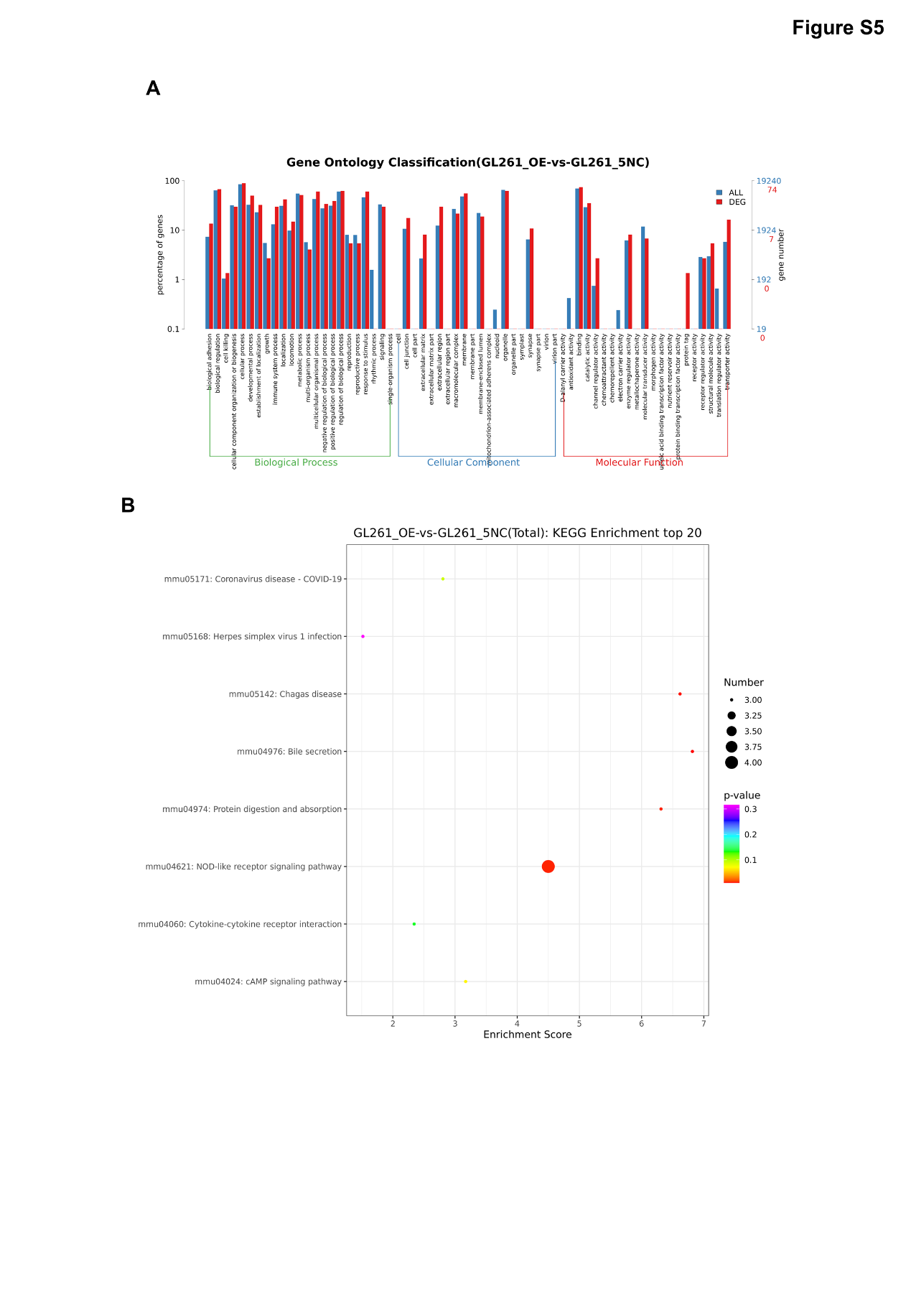


**Fig. S5**: (A) GO enrichment analysis of differentially expressed genes by RNA-sequencing analysis (*n*=3). (B) The bubble map of the first 8 differentially expressed pathways by KEGG enrichment analysis (*n*=3).


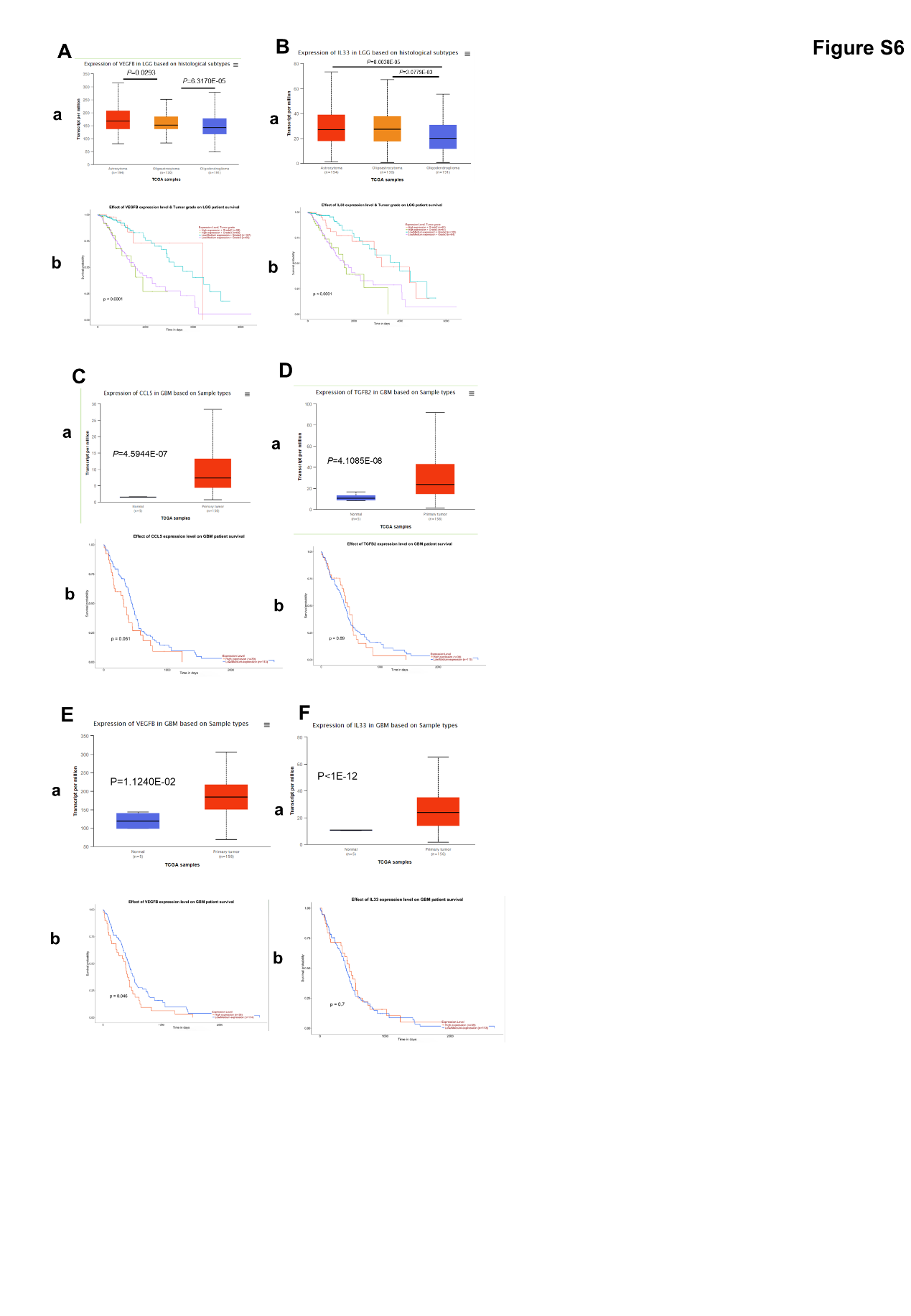


**Fig. S6**: (A,B) IL33 and VEGFβ expression levels in LGG patients (a) and their relationships with survival (b) in TCGA (From http://ualcan.path.uab.edu/). (C-F) CCL5, TGFβ2, IL33, and VEGFβ expression levels in GBM patients (a) and their relationships with survival (b) in TCGA (From http://ualcan.path.uab.edu/).


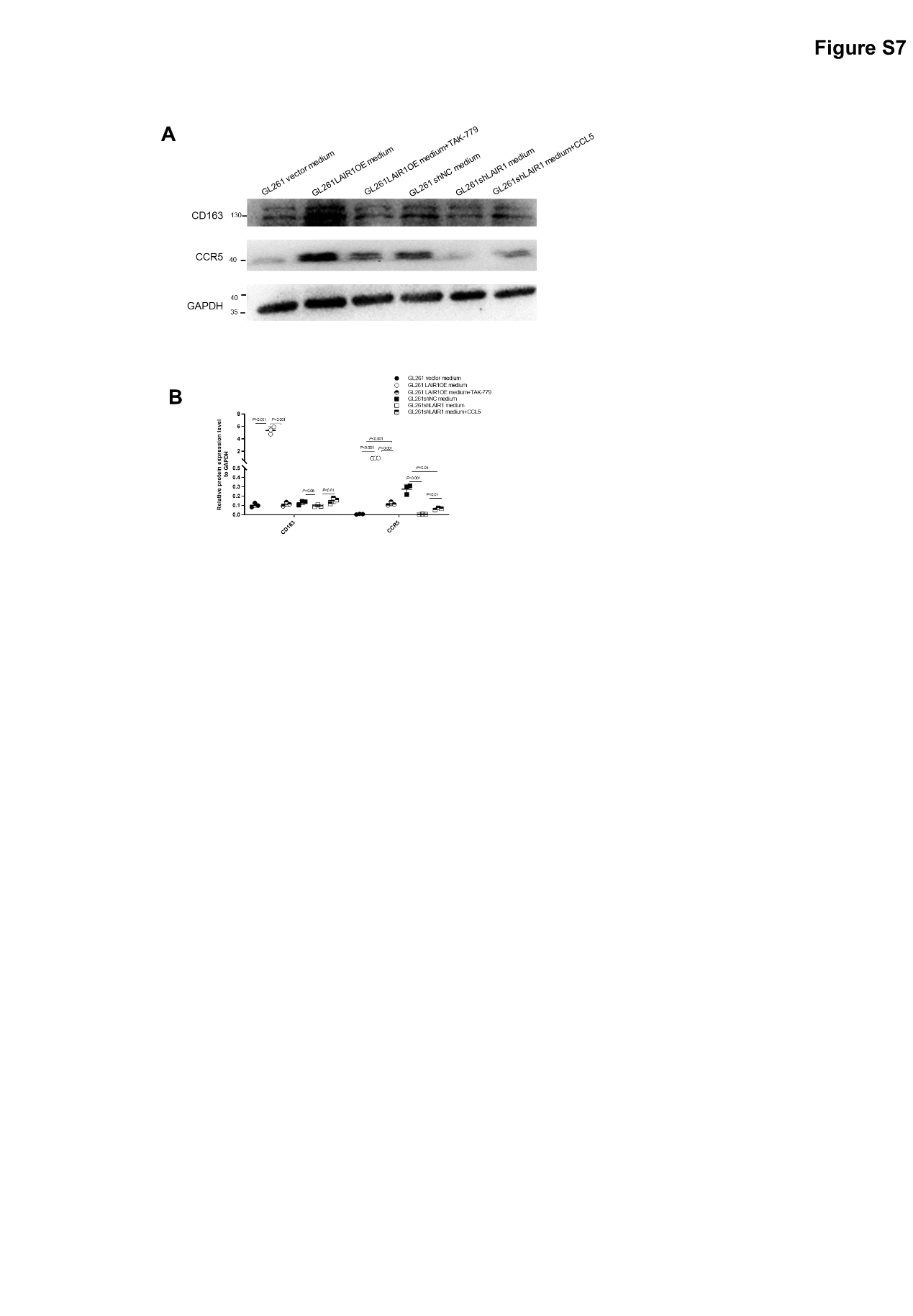


**Fig. S7:** CCR5 expression levels in GBM (A) and LGG patients (B) and their relationships with survival of the patients from TCGA (http://ualcan.path.uab.edu/).

Expression of CD163 and CCR5 in BV2 cells cultured using the medium of LAIR1OE or shLAIR1 GL261 cells in the presence of CCL5 (10 ng/mL) or CCR5 antagonist TAK-779 (1.2 nM) for 48 h by Western blot and quantification (*n*=3). The data were expressed as average ±SD. *P* values were labeled in each figure.

**Table S1. Correlation between LAIR1 expression and clinicopathological characteristics**

|  | variables | LAIR1 expression | | total | *χ2* | *P* value |
| --- | --- | --- | --- | --- | --- | --- |
|  |  | low | high |  |  |  |
| Age(year) |  |  |  |  | 6.778 | 0.009 |
|  | ≤40 | 65 | 12 | 77 |  |  |
|  | >40 | 56 | 28 | 84 |  |  |
| Gender |  |  |  |  | 0.581 | 0.466 |
|  | Female | 45 | 18 | 63 |  |  |
|  | Male | 76 | 23 | 99 |  |  |
| Grade |  |  |  |  | 10.415 | 0.001 |
|  | Ⅰ/Ⅱ | 94 | 21 | 115 |  |  |
|  | Ⅲ/Ⅳ | 27 | 20 | 47 |  |  |

**Table S2. Materials and regents used in this study**

| Name | Company | Location |
| --- | --- | --- |
| DMEM medium | Corning | Corning, NY, USA |
| Fatal bovine serum (04102-1) | Biological Industries | Kibbutz Beit Haemek, Israel |
| Penicillin-streptomycin(15140122) | GIBCO, Thermo Fisher Scientific, Inc. | Waltham, MA, USA |
| Phosphate-Buffered Saline (10X) | Solarbio | Beijing, China |
| CellTiter 96® AQueous One Solution Cell Proliferation Assay | Promega | Madison, WI, USA |
| Transwell plate | Corning | Corning, NY, USA |
| Dishes | Corning | Corning, NY, USA |
| Hanks’ Balanced Salt solution | Sigma Aldrich | St.Louis, MO, USA |
| RIPA Lysis Buffer (Strong) (HY-K1001) | MedChemExpress | Shanghai, China |
| Protease Inhibitor Cocktail (P8340) | Sigma Aldrich | St.Louis, MO, USA |
| Phosphatase Inhibitor Cocktail (P0044) | Sigma Aldrich | St.Louis, MO, USA |
| CelLytic™ NuCLEAR™ extraction kit (NXTRACT-1KT) | Sigma Aldrich | St.Louis, MO, USA |
| Albumin Bovine V | Solarbio | Beijing, China |
| TBST Buffer (20X) | Solarbio | Beijing, China |
| Annex V/PI staining kit | Solarbio | Beijing, China |
| Immobiloin-PSQ Transfer Membranes | Merck Millipore Ltd. | Billerica, MA, USA |
| LAIR1 Antibody (#60061) | Cell Signaling Technology | Danvers, MA, USA |
| LAIR1 Antibody(#[sc-398141](https://www.scbt.com/zh/p/lair-1-antibody-f-5?requestFrom=search)) | Santa Cruz | Santa Cruz Biotechnology, Inc., Shanghai, China |
| Phospho-FAK Antibody (#8556) | Cell Signaling Technology | Danvers, MA, USA |
| FAK Antibody (#3285P) | Cell Signaling Technology | Danvers, MA, USA |
| CDK4 Antibody (ab68266) | Abcam | Waltham, MA, USA |
| CDK6 Antibody (ab241554) | Abcam | Waltham, MA, USA |
| Cyclin D1 Antibody (#55506) | Cell Signaling Technology | Danvers, MA, USA |
| TGFβ Antibody (#3T11S) | Cell Signaling Technology | Danvers, MA, USA |
| Bcl-2 Antibody (#15071) | Cell Signaling Technology | Danvers, MA, USA |
| Bax Antibody (#R380709) | Chengdu Zen-Bioscience Co., Ltd. | Chengdu, Sichuan, China |
| Ki-67 Antibody (#9129S) | Cell Signaling Technology | Danvers, MA, USA |
| CCL5/RANTES Antibody (#36467) | Cell Signaling Technology | Danvers, MA, USA |
| Goat anti-rabbit IgG (#ab205718) | Abcam | Cambridge, MA, USA |
| Goat anti-mouse IgG (#ab6789) | Abcam | Cambridge, MA, USA |
| Anti-GAPDH (#3683) | Cell Signaling Technology | Danvers, MA, USA |
| SHP1 Antibody (R25714) | Chengdu Zen-Bioscience Co., Ltd. | Chengdu, Sichuan, China |
| SHP2 Antibody (381305) | Chengdu Zen-Bioscience Co., Ltd. | Chengdu, Sichuan, China |
| Flag-tag Mouse mAb (700002) | Chengdu Zen-Bioscience Co., Ltd. | Chengdu, Sichuan, China |
| CD163 Antibody (222138) | Chengdu Zen-Bioscience Co., Ltd. | Chengdu, Sichuan, China |
| CD80 Antibody (516039) | Chengdu Zen-Bioscience Co., Ltd. | Chengdu, Sichuan, China |
| CD11b Antibody (380675) | Chengdu Zen-Bioscience Co., Ltd. | Chengdu, Sichuan, China |
| APC anti-mouse CD80 Ab (E-AB-F0992E) | Elabscience Biotechnology Co.,Ltd | Wuhan, China |
| APC anti-mouse CD163Ab (E-AB-F1295E) | Elabscience Biotechnology Co.,Ltd | Wuhan, China |
| PE anti-mouse LAIR1 mAb (12-3501-82) | Elabscience Biotechnology Co.,Ltd | Wuhan, China |
| FITC Anti-Mouse CD11b Ab (E-AB-F1081C) | Elabscience Biotechnology Co.,Ltd | Wuhan, China |
| TRIzol (#15596026) | Thermo Fisher Scientific Inc. | Waltham, MA, USA |
| Revert Aid First Strand cDNA Synthesis Kit (K1622) | Thermo Fisher Scientific Inc. | Waltham, MA, USA |
| DyNAmo ColorFlash SYBR Green qPCR kit (F-416) | Thermo Fisher Scientific Inc. | Waltham, MA, USA |
| ReverTra Ace qPCR RT Kit | Toyobo | Shanghai, China |
| FG, MICROPLATE, LHS 96 WELL | Applied Biosystems | Foster City, CA, USA |
| OPTICAL ADHESIVE COVERS, CAP | Applied Biosystems | Foster City, CA, USA |
| PCR strip tubes | Axygen | Tewksbury, MA, USA |
| Genes Primers | Sangon Biotech | Shanghai, China |
| TRizol Reagent | Sigma Aldrich | St.Louis, MO, USA |
| Opti-MEM I Reduced Serum Medium | Invitrogen | Carlsbad, CA, USA |
| BCA Protein Assay Kit (#C503021) | Sangon Biotech Co., Ltd. | Shanghai, China |
| SDS-PAGE Gel Quick Preparation Kit (P0012AC) | Beyotime Biotechnology | Shanghai, China |
| Immobilon^TM^ Western Chemiluminescent HRP Substate (WBKLS0500) | Merck Millipore Ltd. | Billerica, MA, USA |
| BeaverBeads™ Protein A/G Immunoprecipitation Kit (22202-100) | Beaver Biosciences Inc. | Guangzhou, China |
| Lipofectamin 3000 Reagent | Invitrogen | Carlsbad, CA, USA |
| human glioma tissue chip (HBraG180Su01) | Shanghai Outdo Biotech Co., LTD | Shanghai, China |
| LV5-LAIR1 overexpression [plasmid](https://fanyi.so.com/?src=onebox#plasmid) (190721BZ) | GenePharma | Suzhou, China |
| LV5-NC(G23YZ) | GenePharma | Suzhou, China |
| LAIR1 shRNA | Shanghai Genechem Co. | Shanghai, China |
| Y12765 LAIR1Y251281F (2022 46379) | GenePharma | Suzhou, China |
| Y12764 LAIR1 wt (2022 46379) | GenePharma | Suzhou, China |
| Annexin V-FITC/PI Apoptosis Kit (E-CK-A211) | Beyotime Biotechnology | Shanghai, China |
| One-step TUNEL In Situ Apoptosis Kit (Green, FITC) E-CK-A320 | Beyotime Biotechnology | Shanghai, China |
| Hematoxylin-Eosin staining (G1120) | Solarbio | Shanghai, China |
| Trypan Blue Stain solution,0.4% ([C0040](https://www.solarbio.com/goods-242.html)) | Solarbio | Shanghai, China |
| Paraformaldehyde,4% (P1110) | Solarbio | Shanghai, China |
| Mouse IL33 ELISA KIT (SEKH-0028) | Solarbio | Shanghai, China |
| [Mouse VEGF ELISA KIT](https://www.solarbio.com/goods-38400.html) ([SEKH-0039](https://www.solarbio.com/goods-38400.html)) | Solarbio | Shanghai, China |
| [Mouse CCL5 ELISA KIT](https://www.solarbio.com/goods-38409.html) ([SEKH-0043](https://www.solarbio.com/goods-38409.html)) | Solarbio | Shanghai, China |
| [Mouse TGFβ2 ELISA KIT](https://www.solarbio.com/goods-38451.html) ([SEKH-0036](https://www.solarbio.com/goods-38451.html)) | Solarbio | Shanghai, China |
| Palbociclib (PD-0332991) | MedChemexpress CO., Ltd | Princeton, NJ, USA |
| Adezmapimod (SB 203580) | MedChemexpress CO., Ltd | Princeton, NJ, USA |
| JNK inhibitor (SP600125) | MedChemexpress CO., Ltd | Princeton, NJ, USA |
| ERK inhibitor (PD98059) | MedChemexpress CO., Ltd | Princeton, NJ, USA |
| AKT inhibitor (MK2206) | MedChemexpress CO., Ltd | Princeton, NJ, USA |
| mTOR inhibitor (INK-128) | MedChemexpress CO., Ltd | Princeton, NJ, USA |
| PI3K inhibitor (HY-101115) | MedChemexpress CO., Ltd | Princeton, NJ, USA |
| caspase inhibitor (Z-VAD-FMK) | MedChemexpress CO., Ltd | Princeton, NJ, USA |
| AMPK inhibitor (BML275) | MedChemexpress CO., Ltd | Princeton, NJ, USA |
| FAK inhibitor (Y15) | MedChemexpress CO., Ltd | Princeton, NJ, USA |
| FAK inhibitor (GSK215) | MedChemexpress CO., Ltd | Princeton, NJ, USA |
| Super Plus™ High Sensitive and Rapid Immunohistochemical Kit (pH9.0) (E-IR-R220) | Solarbio | Shanghai, China |
| Opal 4-Color Manual IHC Detection Kit (NEL810001KT) | PerkinElmer | MA, USA |
| Prolong Antifade fluorescence mounting medium (P36984) | Invitrogen | Carlsbad, CA, USA |

**Table S3. Primers and constructs for PCR and cloning**

| Study | Names | Sequences |
| --- | --- | --- |
| PCR | F-*Lair1* | CTTTGTCTTTCCGCCCTTCTG |
|  | R-*Lair1* | GCCTGTCATCTGCAACTATGT |
|  | F-*Ccl5* | GCTGCTTTGCCTACCTCTCC |
|  | R-*Ccl5* | TCGAGTGACAAACACGACTGC |
|  | F-*Il33* | GGATCCGATTTTCGAGAGCTTAAACAT |
|  | R-*Il33* | GCGGCCGCATGAGACCTAGAATGAAGT |
|  | F-*Vegfβ* | GGAGAAGAATGTGGTTAAGATCTGTGA |
|  | R-V*egfβ* | ACACATCGCTCTGAATTGTGTATACT |
|  | F-*Tgfβ* | CTTCGACGTGACAGACGCT |
|  | R-*Tgfβ* | GCAGGGGCAGTGTAAACTTATT |
|  | F-*Il12* | TGCCGCTACTTCTCCTCAG |
|  | R-*Il12* | ACTTCATGGTTCGGTTCCCAA |
|  | F-*Tnfα* | GGAACACGTCGTGGGATAATG |
|  | R- *Tnfα* | GGCAGACTTTGGATGCTTCTT |
|  | F-*Il10* | TTGTCGCGTTTGCTCCCATT |
|  | R- *Il10* | GAAGGGCTTGGCAGTTCTG |
|  | F-*Gapdh* | GGTGCTGAGTATGTCGTGGAGTCTA |
|  | R-*Gapdh* | AAAGTTGTCATGGATGACCTTGG |
|  | F-*Ccnd1* | CTGGATGCTGGAGGTCTGG |
|  | R-*Ccnd1* | GGCAGTCAAGGGAATGGTCT |
| SHP2 cloning | F-*Shp2* (for amplification) | ACCTGTATTTTCAGGGATCCGGAGGACGTATAAATGCTGCTGAAAT |
|  | R-*Shp2* (for amplification | GCTTTGTTAGCAGCCGGATCCTTATAGTGTTTCAATATAATG |
