# Supplementary material for: LAIR1 drives glioma progression by nuclear focal adhesion kinase dependent expressions of cyclin D1 and immunosuppressive chemokines/cytokines
Source: Cell Death Dis. 2023 Oct 16;14(10):684. doi: 10.1038/s41419-023-06199-9 (PMC10579300; doi:10.1038/s41419-023-06199-9)
Supplement: Supplementary file 2 — Original WB [file 41419_2023_6199_MOESM2_ESM.pdf]

Figure 2A

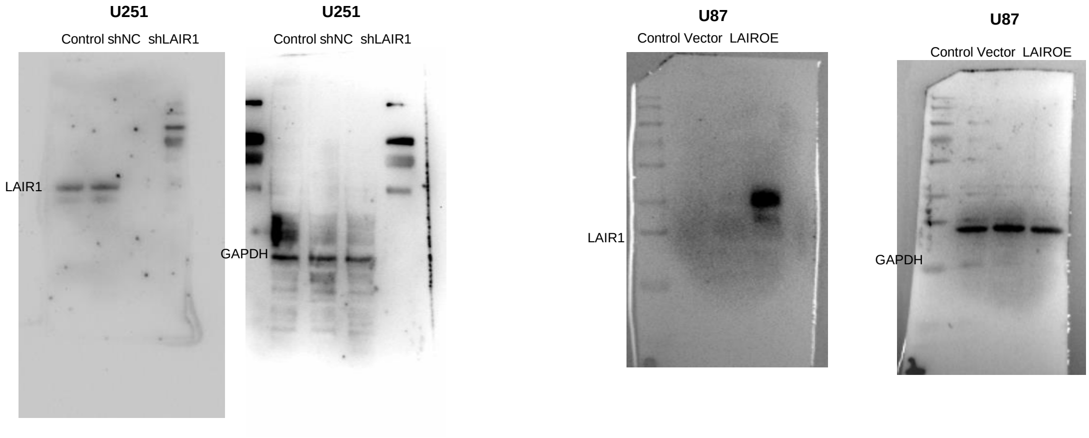

Figure 2H

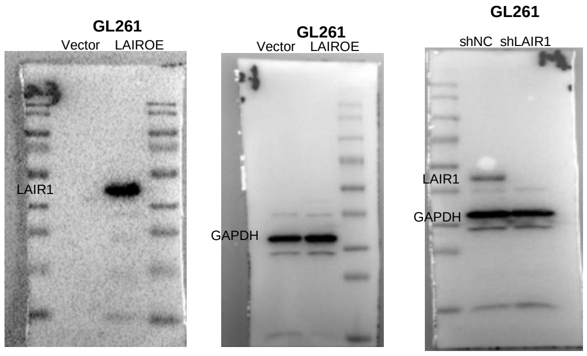

Figure3H

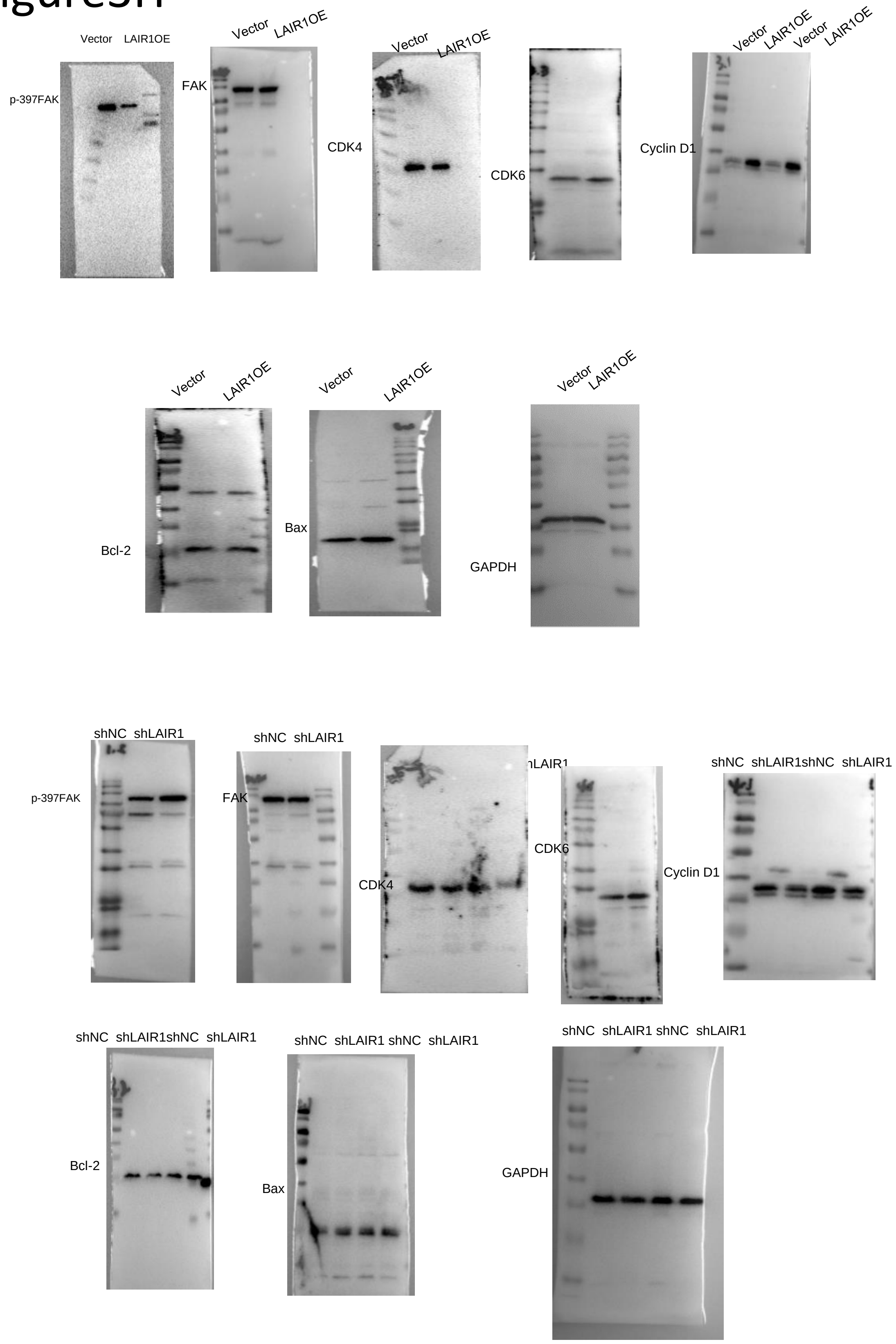
Vector LAIR1OE
p-397FAK
Vector LAIR1OE
FAK
Vector LAIR1OE
CDK4
CDK6
Vector LAIR1OE Vector LAIR1OE
Cyclin D1
Vector LAIR1OE
Bcl-2
Vector LAIR1OE
Bax
Vector LAIR1OE
GAPDH
shNC shLAIR1
p-397FAK
shNC shLAIR1
FAK
CDK4
CDK6
shNC shLAIR1shNC shLAIR1
Cyclin D1
shNC shLAIR1shNC shLAIR1
Bcl-2
shNC shLAIR1 shNC shLAIR1
Bax
shNC shLAIR1 shNC shLAIR1
GAPDH

Figure3I

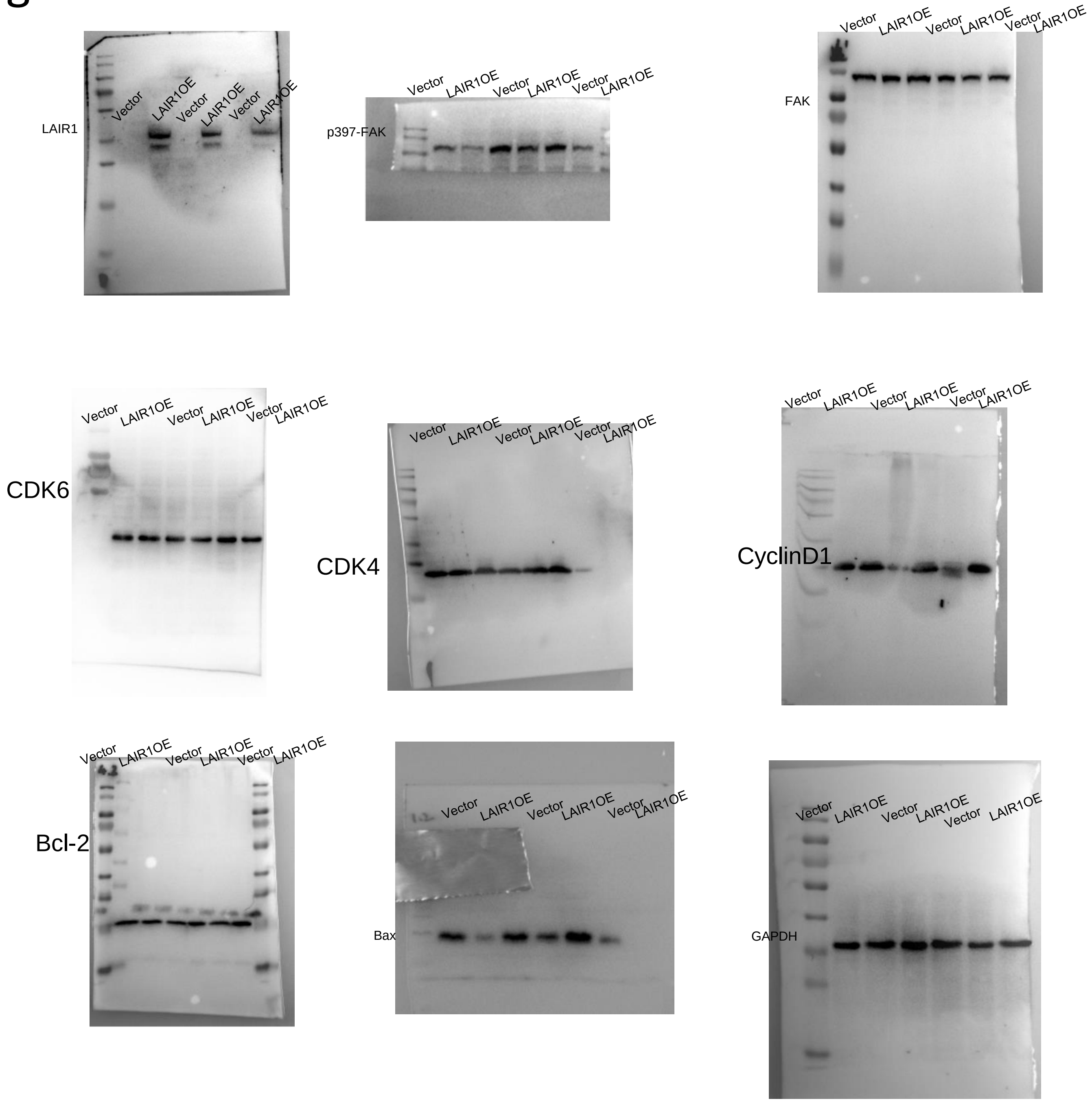
LAIR1
Vector LAIR1OE Vector LAIR1OE Vector LAIR1OE
p397-FAK
Vector LAIR1OE Vector LAIR1OE Vector LAIR1OE
FAK
Vector LAIR1OE Vector LAIR1OE Vector LAIR1OE
CDK6
Vector LAIR1OE Vector LAIR1OE Vector LAIR1OE
CDK4
Vector LAIR1OE Vector LAIR1OE Vector LAIR1OE
CyclinD1
Vector LAIR1OE Vector LAIR1OE Vector LAIR1OE
Bcl-2
Vector LAIR1OE Vector LAIR1OE Vector LAIR1OE
Bax
Vector LAIR1OE Vector LAIR1OE Vector LAIR1OE
GAPDH
Vector LAIR1OE Vector LAIR1OE Vector LAIR1OE

Fig 4C

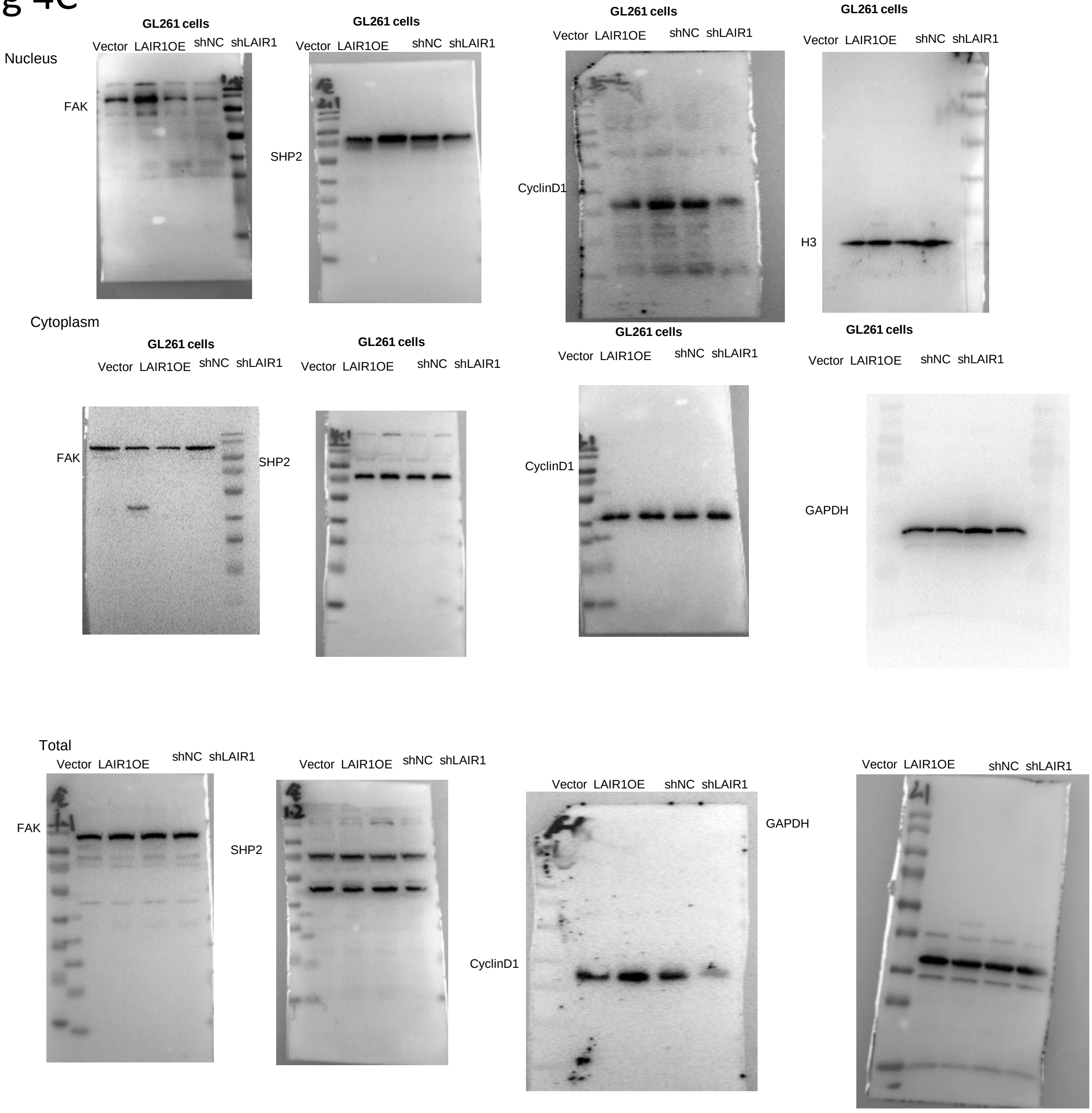
Nucleus
GL261 cells
Vector LAIR1OE shNC shLAIR1
FAK
SHP2
CyclinD1
H3
Cytoplasm
GAPDH
Total

Fig4G

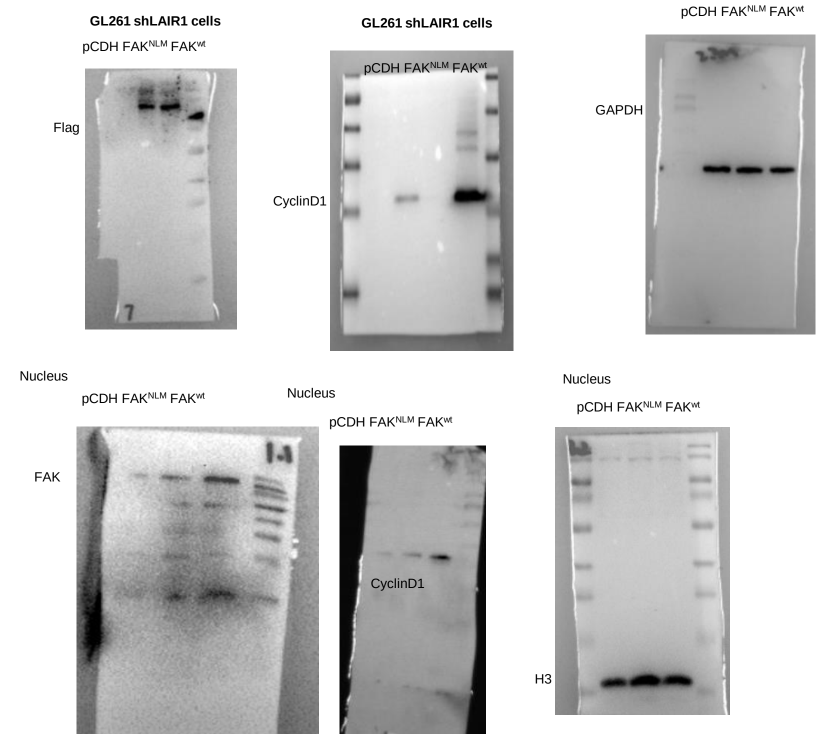
GL261 shLAIR1 cells
pCDH FAK^{NLM} FAK^{wt}
Flag
CyclinD1
GAPDH
Nucleus
FAK
H3

Fig5I

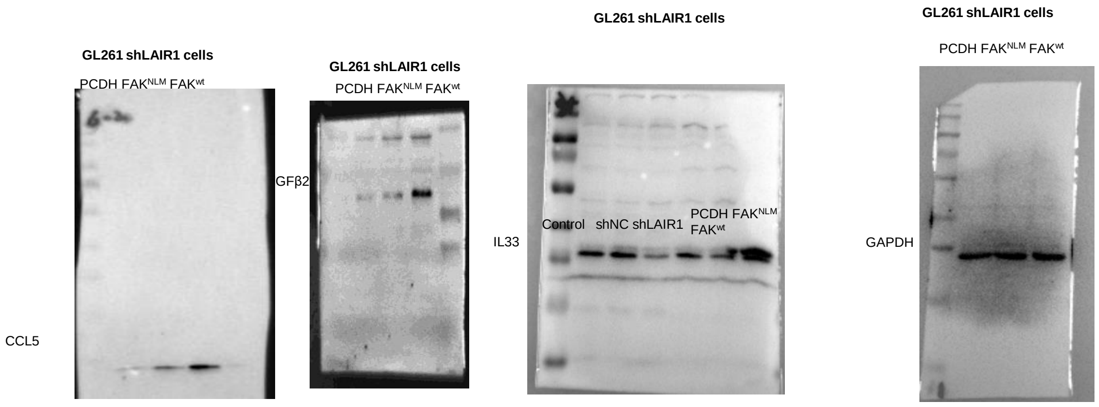
GL261 shLAIR1 cells
PCDH FAK^{NLM} FAK^{wt}
CCL5
GL261 shLAIR1 cells
PCDH FAK^{NLM} FAK^{wt}
GFβ2
GL261 shLAIR1 cells
IL33
Control shNC shLAIR1 PCDH FAK^{NLM} FAK^{wt}
GL261 shLAIR1 cells
PCDH FAK^{NLM} FAK^{wt}
GAPDH

Fig 7A

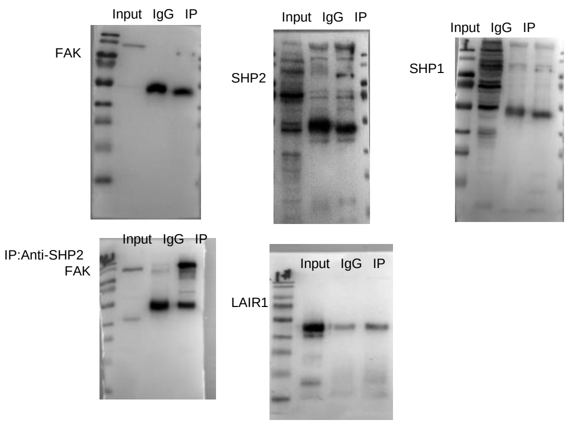

Fig 7C

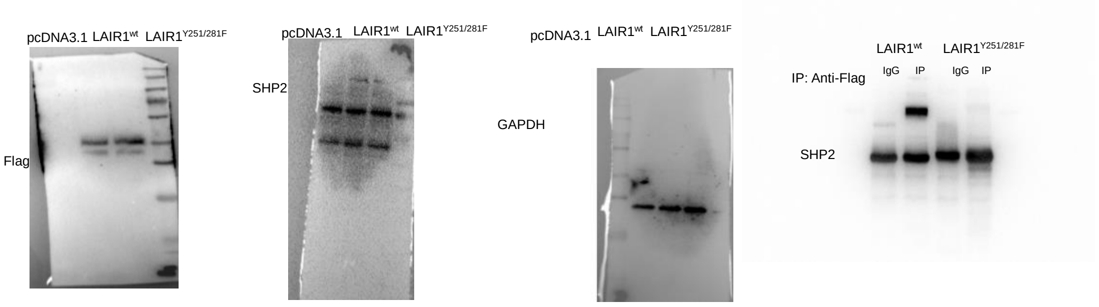

Fig 7D

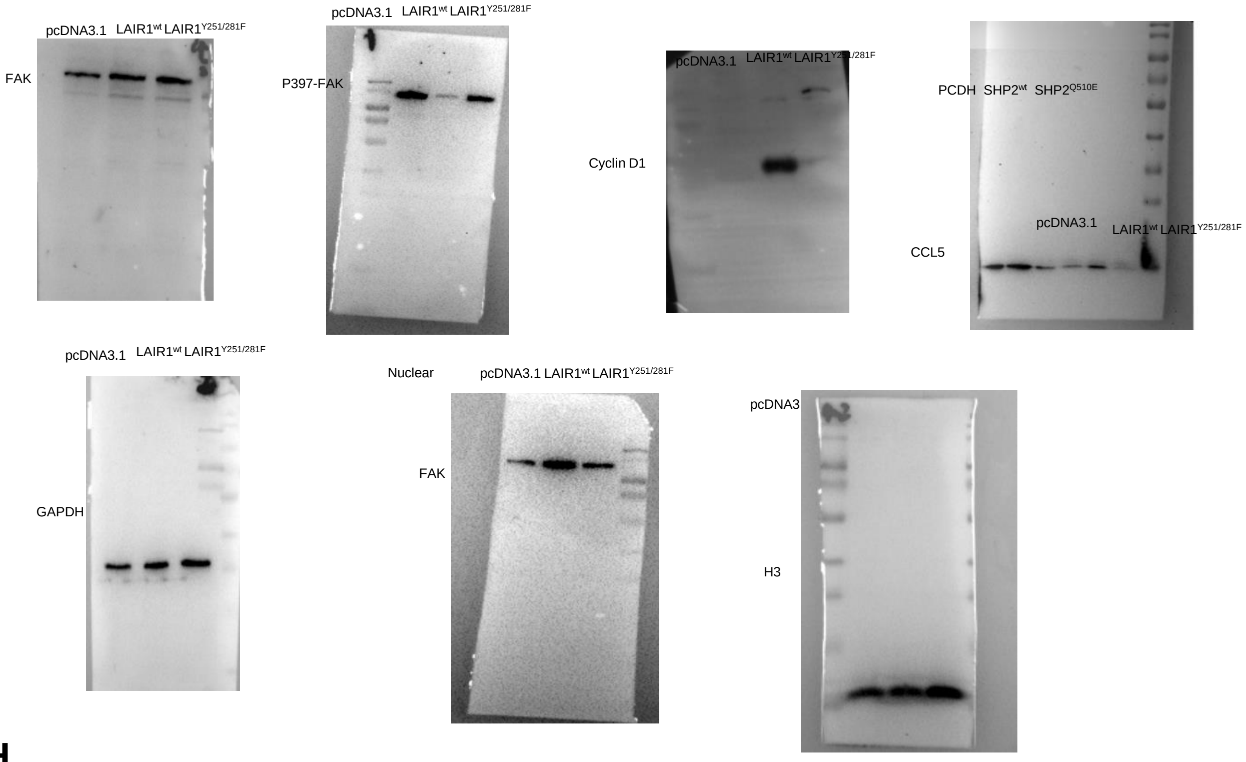

Fig 7H

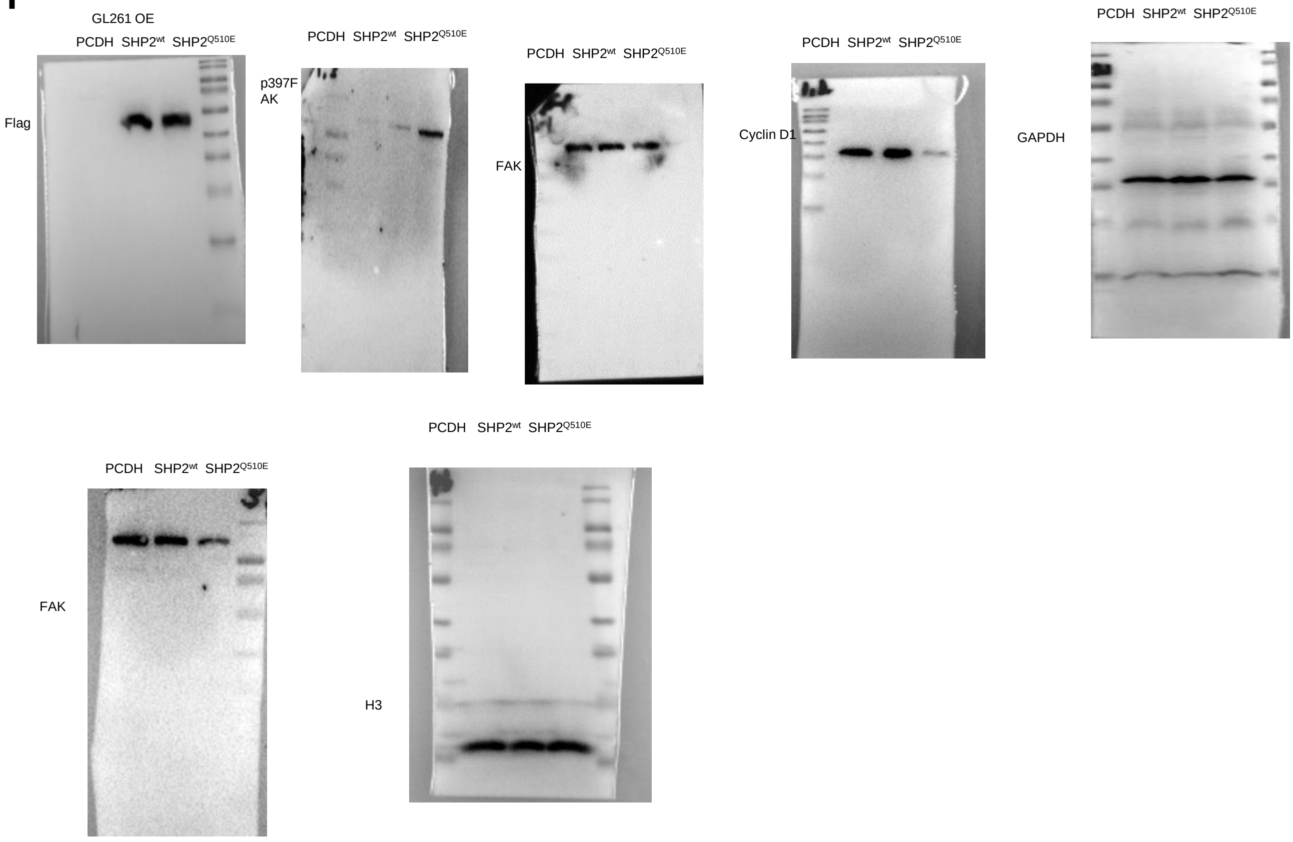

Figure S2B

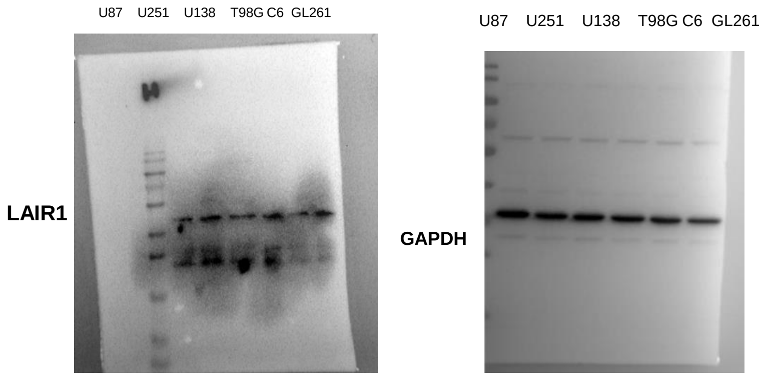

Figure S2C

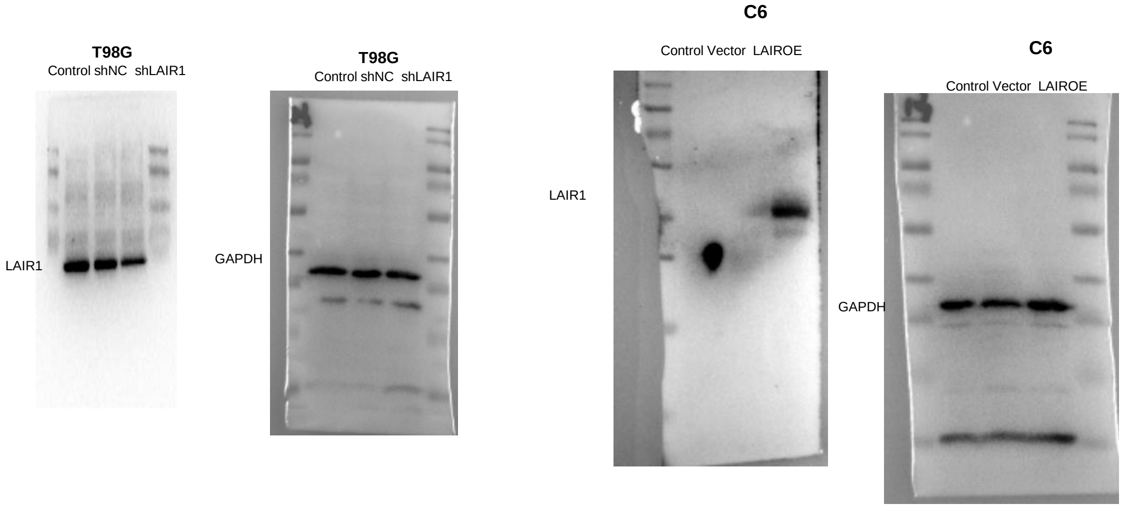

Fig S4

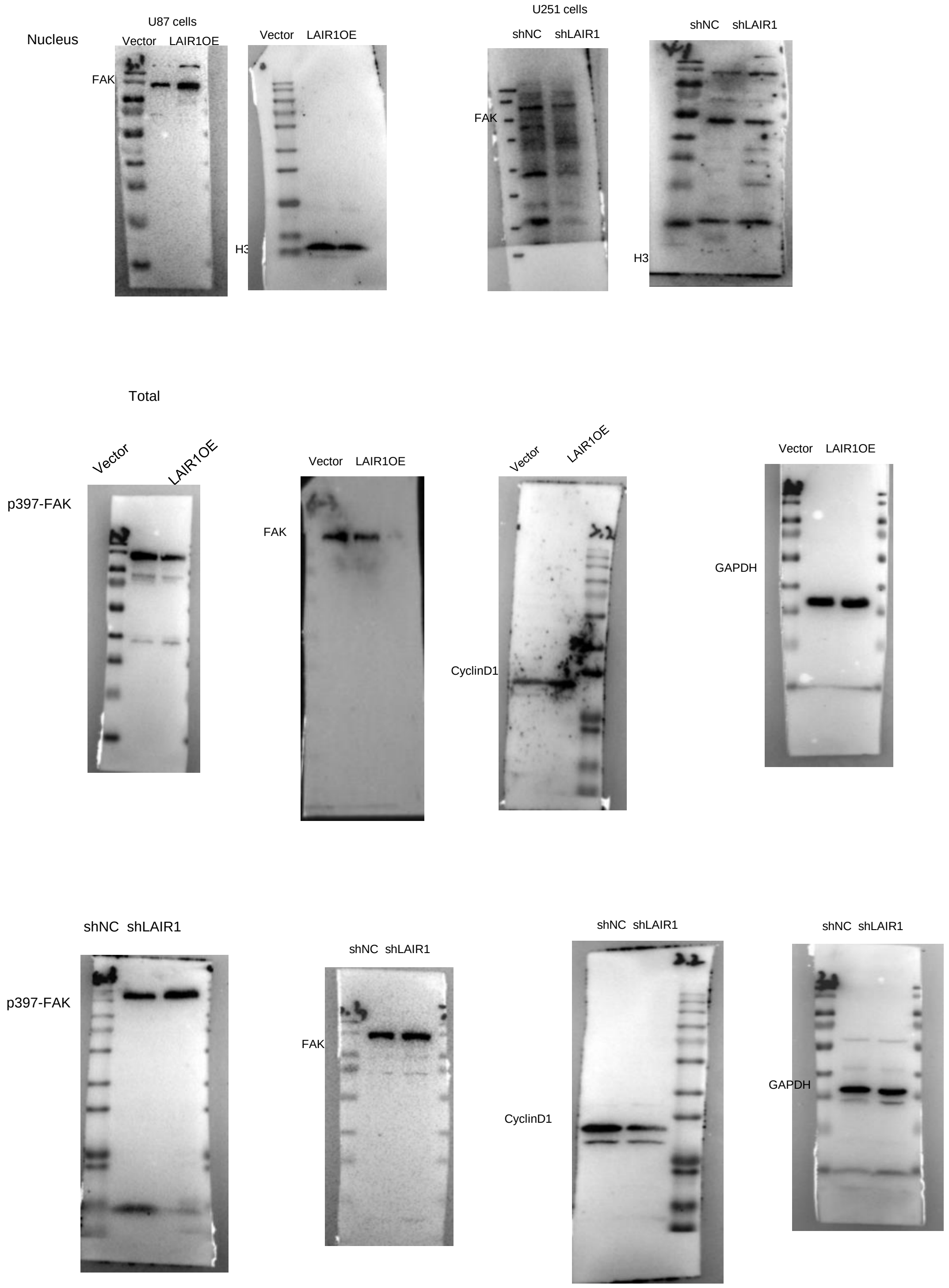

Nucleus
U87 cells
Vector LAIR1OE
FAK
Vector LAIR1OE
H3
U251 cells
shNC shLAIR1
FAK
shNC shLAIR1
H3
Total
Vector
LAIR1OE
p397-FAK
Vector LAIR1OE
FAK
Vector
LAIR1OE
CyclinD1
Vector LAIR1OE
GAPDH
shNC shLAIR1
p397-FAK
shNC shLAIR1
FAK
shNC shLAIR1
CyclinD1
shNC shLAIR1
GAPDH

Fig 6F

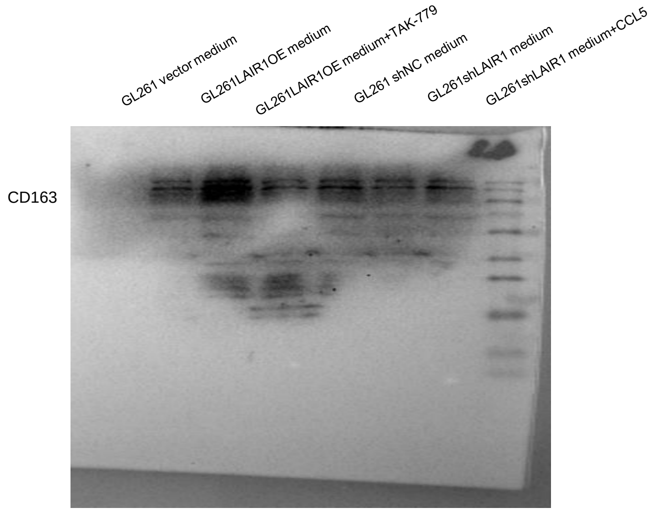
GL261 vector medium
GL261LAIR1OE medium
GL261LAIR1OE medium+TAK-779
GL261 shNC medium
GL261shLAIR1 medium
GL261shLAIR1 medium+CCL5
CD163

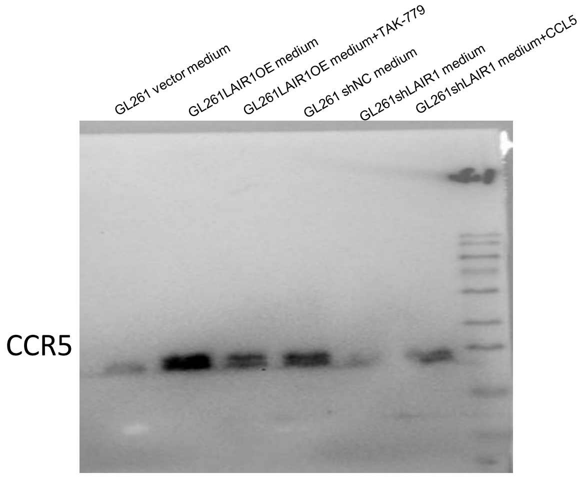
GL261 vector medium
GL261LAIR1OE medium
GL261LAIR1OE medium+TAK-779
GL261 shNC medium
GL261shLAIR1 medium
GL261shLAIR1 medium+CCL5
CCR5

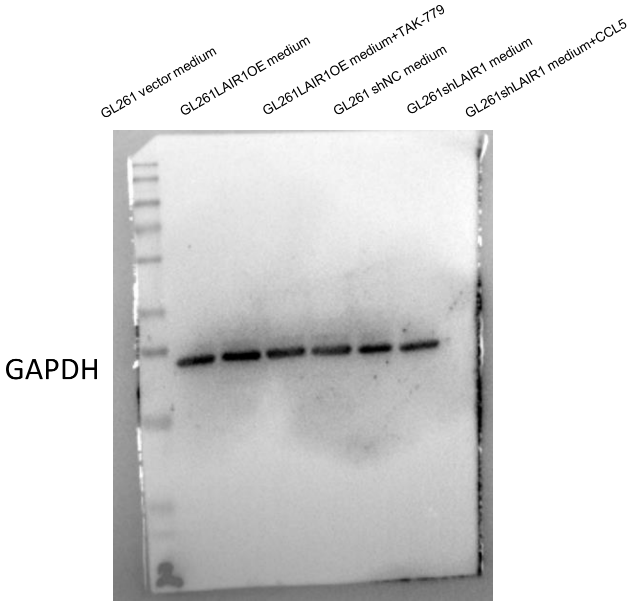
GL261 vector medium
GL261LAIR1OE medium
GL261LAIR1OE medium+TAK-779
GL261 shNC medium
GL261shLAIR1 medium
GL261shLAIR1 medium+CCL5
GAPDH
